# Perinatal exposure to diets with different n-6:n-3 fatty acid ratios affects olfactory tissue fatty acid composition

**DOI:** 10.1038/s41598-020-67725-9

**Published:** 2020-07-01

**Authors:** Spiro Khoury, Vanessa Soubeyre, Stéphanie Cabaret, Laetitia Merle, Stéphane Grégoire, Nicolas Deprêtre, David Jarriault, Xavier Grosmaitre, Lionel Bretillon, Olivier Berdeaux, Niyazi Acar, Anne Marie Le Bon

**Affiliations:** Centre des Sciences du Goût et de l’Alimentation, AgroSup Dijon, CNRS, INRAE, Université Bourgogne Franche-Comté, 21000 Dijon, France

**Keywords:** Biochemistry, Chemical biology, Neuroscience

## Abstract

The olfactory mucosa (OM) and the olfactory bulb (OB) are responsible for the detection and processing of olfactory signals. Like the brain and retina, they contain high levels of n-3 and n-6 polyunsaturated fatty acids (PUFAs), which are essential for the structure and function of neuronal and non-neuronal cells. Since the influence of the maternal diet on olfactory lipid profiles of the offspring has been poorly explored, we examined the effects of feeding mice during the perinatal period with diets containing an adequate linoleic acid level but either deficient in α-linolenic acid (ALA) or supplemented in n-3 long-chain PUFAs on the lipid composition of dams and weaning offspring olfactory tissues. In both the OM and OB, the low n-3 ALA diet led to a marked reduction in n-3 PUFAs with a concomitant increase in n-6 PUFAs, whereas consumption of the high n-3 PUFA diet reduced n-6 PUFAs and increased n-3 PUFAs. Structural analysis showed that the molecular species profiles of the main phospholipid classes of olfactory tissues from weaning pups were markedly affected by the maternal diets. This study demonstrates that the PUFA status of olfactory tissues is sensitive to diet composition from the early stages of development.

## Introduction

Polyunsaturated fatty acids (PUFAs) are essential for the structure and functioning of neuronal and non-neuronal cells found in nervous tissues such as the brain and retina. They are particularly involved in neuronal survival, neurogenesis, synaptic function and regulation of inflammation^1–3^. PUFAs are found in different lipid species, mainly in membrane phospholipids (PLs). The most abundant PUFAs in the mammalian brain and retina are docosahexaenoic acid (DHA, C22:6n-3), an n-3 PUFA, and arachidonic acid (AA, C20:4n-6), an n-6 PUFA^4–6^. DHA is the major long-chain derivative of the precursor of the n-3 PUFAs, α-linolenic acid (ALA, C18:3n-3), whereas AA is formed from the precursor of the n-6 series, linoleic acid (LA, C18:2n-6). Despite the endogenous capacity of humans to synthesize n-3 PUFAs from their precursor, the rate of conversion is considered to be too low to significantly participate in the tissue levels of n-3 PUFAs^7^, and therefore, humans rely on adequate dietary intake.

Most PUFAs rapidly accumulate in the brain and retina during the later stages of gestation and early postnatal life via placental transfer and maternal milk. The incorporation of PUFAs in the cell membranes during the perinatal period contributes to the functional maturation of nervous tissues. Animal studies have provided evidence for the importance of maternal PUFA intake for the neurological development of offspring. Hence, a dietary supply of n-3 PUFAs during pregnancy and lactation promotes cognitive development, while an n-3 PUFA deficit impairs it^8–12^. Similarly, the perinatal dependence of the retina on the n-3 PUFA supply has been demonstrated for several decades^9^. A deficiency in retinal DHA subsequent to dietary restriction of n-3 PUFAs during gestation and early postnatal life alters the fine-tuning of visual topography in rodents^13^ as well as molecular events involved in visual transduction^14,15^.

In mammals, the olfactory system consists of a variety of specialized neurons that are found in the olfactory mucosa (OM), the olfactory bulb (OB), and other higher order olfactory centres, such as the piriform cortex and the orbitofrontal cortex^16^. The OM is located in the upper region of the nasal cavity. It comprises the olfactory epithelium, which is composed of multiple cell types, including olfactory sensory neurons (OSNs), and the underlying lamina propria. The dendrites of OSNs project toward the mucus layer covering the olfactory epithelium, where they protrude cilia containing odourant receptors. Binding of odourant molecules to receptors triggers a signal transduction cascade that leads to the generation of action potentials that are then conveyed to the OB. The OB is the first brain region in which odour information is processed. At this level, olfactory signals are processed by interneurons (mainly by periglomerular cells and granular cells) before being exported to higher centres of the brain by output neurons (mitral cells and tufted cells).

The lipid composition of the mammalian OM and OB shares common features with that of the brain and retina. We have shown that rodent OM contains a high proportion of PUFAs, with n-3 and n-6 PUFAs accounting for 13% and 23% of total fatty acids (FAs), respectively^17^. N-3 and n-6 PUFAs are found at similar levels in rat OB (20% and 16%, respectively)^18^. DHA and AA are the major n-3 and n-6 PUFAs identified in both tissues. These PUFAs are found in different PL groups, mainly in phosphatidylcholine (PC), phosphatidylethanolamine (PE), phosphatidylinositol (PI) and phosphatidylserine (PS)^17,18^.

Little is known about the impact of PUFAs present in the diet on the PUFA content of olfactory tissues. Dietary depletion of n-3 PUFAs during the prenatal and postnatal periods for 2 generations was reported to decrease PS accumulation^19^ and to significantly increase the C22:5n-6 level in the rat OB^18^. On the other hand, ALA or ALA plus DHA supplementation from conception until postnatal day 70 resulted in a significant enhancement of DHA levels in the OB^20^. Altogether, these findings suggest that the PUFA composition of the mammalian OB can be affected by the maternal diet during development. As far as the OM is concerned, to our knowledge, the influence of PUFA dietary intake on the composition of this tissue has not yet been assessed.

The present study aimed to assess the effects of feeding diets with different n-6:n-3 PUFA ratios to mice during the perinatal period on the lipid composition of olfactory tissues from dams and their offspring. To this end, we analysed the FA composition of the dam and pup OM and OB at weaning as well as that of dam milk. In addition, we characterized the molecular species in each PL class (including plasmalogens) extracted from the OM and OB of 3-week-old pups.

## Results

### Dam tissues

#### Fatty acid composition of maternal milk

Maternal milk was collected 15 days after the pups were born (P15). At this time, dams were fed with the experimental diets (CON, LOW or HIGH diets; Table 1) for 5 weeks. There was no significant difference in the total lipid content of milk between the CON, LOW and HIGH groups (16.9 ± 1.1%, 17.7 ± 2.4%, and 17.2 ± 0.80%, respectively). Compared to the CON group, milk from the LOW dams contained significantly less n-3 PUFAs (− 73%) (Table 2*).* Although the total n-6 PUFA content was not modified compared to that of CON, a small but significant increase in C20:4n-6 and C22:4n-6 was observed in milk from the LOW group. Hence, the n-6:n-3 ratio in the LOW milk was higher than that in the CON milk (23.15 ± 5.08% versus 5.89 ± 0.07%, respectively). In contrast, the n-6:n-3 ratio of the HIGH milk was significantly reduced compared to the CON group (3.41 ± 0.10% versus 5.89 ± 0.07%, respectively). This effect was due to the strong increase in n-3 PUFAs (C20:5n-3; C22:5n-3; and C22:6n-3) compared to CON milk. The milk n-6 PUFA content was not altered by the maternal HIGH diet. As a marker of essential PUFA deficiency^21^, Mead acid (C20:3n-9) was significantly increased by + 33% in LOW milk and decreased by half in HIGH milk compared to CON milk.

**Table 1:** Fatty acid composition of the experimental diets (% of total fatty acids).

Fatty acids	Experimental diets		
	CON	LOW	HIGH
12:0	0.15	0.15	0.14
14:0	0.53	0.60	1.32
15:0			0.12
16:0	13.32	16.00	9.44
16:1n-9			0.10
16:1n-7	0.20	0.17	1.19
17:0			0.14
18:0	4.62	4.65	4.31
18:1 t	3.85	3.39	3.22
18:1n-9	54.32	55.81	51.74
18:1n-7	2.09	0.99	2.32
18:2n-6 (LA)	16.49	16.91	17.62
20:0	0.40	0.28	0.45
20:1n-9	0.52	0.17	0.66
18:3n-3 (ALA)	2.84	0.15	3.03
22:0	0.43	0.58	0.50
22:1n-9	0.11		0.13
20:4n-6			0.14
24:0	0.16	0.21	0.15
20:5n-3 (EPA)			1.97
24:1n-9			0.10
22:5n-3 (DPA n-3)			0.19
22:6n-3 (DHA)			1.08
SFAs	19.59	22.44	16.55
MUFAs	61.08	60.52	59.43
PUFAs	19.33	17.05	24.03
Total n-6 PUFAs	16.49	16.91	17.76
Total n-3 PUFAs	2.84	0.15	6.27
n-6:n-3 PUFA ratio	5.80	116.73	2.83

*CON* control diet, *LOW* low n-3 diet, *HIGH* high n-3 diet, *ALA* α-linolenic acid, *DHA* docosahexaenoic acid, *DPA* docosapentaenoic acid, *EPA* eicosapentaenoic acid, *LA* linoleic acid, *MUFAs* monounsaturated fatty acids, *PUFAs* polyunsaturated fatty acids, *SFAs* saturated fatty acids.

**Table 2:** Fatty acid composition of maternal milk samples (% of total fatty acids).

Fatty acids	Maternal milk			
	CON		LOW	HIGH
10:0	7.24 ± 0.29		7.38 ± 1.05	7.72 ± 0.41
12:0	12.64 ± 0.45		13.09 ± 1.84	13.23 ± 0.51
14:0	16.30 ± 0.55		16.68 ± 2.29	17.07 ± 0.49
14:1	0.15 ± 0.02		0.16 ± 0.02	0.18 ± 0.01
15:0	0.08 ± 0.01		0.08 ± 0.01	0.10* ± 0.01
16:0	27.28 ± 0.64		27.71 ± 3.77	26.50 ± 1.11
16:1n-9	0.25 ± 0.01		0.28 ± 0.04	0.20 ± 0.02
16:1n-7	1.61 ± 0.10		1.59 ± 0.24	1.97* ± 0.09
18:0	2.37 ± 0.15		2.49 ± 0.34	2.03 ± 0.11
18:1 t	0.48 ± 0.03		0.52 ± 0.07	0.49 ± 0.03
18:1n-9	22.08 ± 0.87		21.47 ± 3.08	20.41 ± 0.98
18:1n-7	2.15 ± 0.15		1.99 ± 0.31	2.01 ± 0.10
18:2n-6	4.34 ± 0.10		3.88 ± 0.59	4.74 ± 0.20
20:0	0.07 ± 0.01		0.07 ± 0.01	0.07 ± 0.01
20:1n-9	0.92 ± 0.09		1.01 ± 0.15	0.67 ± 0.07
18:3n-3	0.59 ± 0.02		0.09* ± 0.13	0.61 ± 0.03
20:2n-6	0.37 ± 0.03		0.40 ± 0.06	0.36 ± 0.03
20:3n-9	0.09 ± 0.01		0.12* ± 0.02	0.05* ± 0.01
20:3n-6	0.25 ± 0.01		0.28 ± 0.04	0.23 ± 0.02
22:1n-9	0.07 ± 0.01		0.09 ± 0.01	0.06 ± 0.01
20:4n-6	0.21 ± 0.02		0.31* ± 0.04	0.18 ± 0.02
20:5n-3	0.09 ± 0.01		0.07 ± 0.01	0.27* ± 0.02
24:1n-9	0.08 ± 0.01		0.08 ± 0.01	0.07 ± 0.01
22:4n-6	0.09 ± 0.01		0.14* ± 0.02	0.06 ± 0.01
22:5n-3	0.09 ± 0.01		0.02* ± 0.02	0.30* ± 0.01
22:6n-3	0.12 ± 0.01		0.06* ± 0.02	0.44* ± 0.02
SFAs	65.98 ± 1.23		67.50 ± 9.19	66.71 ± 1.44
MUFAs	27.79 ± 1.08		27.16 ± 3.89	26.06 ± 1.17
PUFAs	6.24 ± 0.16		5.36* ± 0.85	7.24* ± 0.28
Total n-6 PUFAs	5.26 ± 0.14		5.01 ± 0.72	5.56 ± 0.24
Total n-3 PUFAs	0.89 ± 0.02		0.24* ± 0.18	1.63* ± 0.06
n-6:n-3 ratio	5.89 ± 0.07		23.15* ± 5.08	3.41* ± 0.10

Data *are* expressed as the mean ± S.E.M. (n = 4–6 mice/group).

*MUFAs* monounsaturated fatty acids, *PUFAs* polyunsaturated fatty acids, *SFAs* saturated fatty acids.

*Values are significantly different from the CON group (Mann–Whitney U test, p < 0.05).

#### Fatty acid composition of the maternal olfactory mucosa and olfactory bulb

The FA profiles of maternal OM and OB were analysed at the end of the lactation period, i.e., in dams fed with the experimental diets for 6 weeks. OM from the LOW dams had significantly higher total n-6 PUFA levels (C20:4n-6, C22:4n-6, and C22:5n-6; + 22%) and lower total n-3 FA levels (- 20%) than the dams of the CON group (Table 3). In contrast, the OM of dams fed the HIGH diet showed increased n-3 PUFA levels (+ 18%) and reduced n-6 PUFA levels (− 12%) compared to the CON group. There was no difference among the 3 groups in the levels of saturated FAs (SFAs), monounsaturated FAs (MUFAs), PUFAs and dimethyl acetals (DMAs), which are residues of plasmalogens. In the OB of dams, the consumption of the LOW diet also resulted in a significant decrease in n-3 PUFAs (− 15%) and in a modest but significant increase in n-6 PUFAs (+ 10%), whereas the HIGH diet enhanced the n-3 PUFA level (+ 15%) and reduced the n-6 PUFA level (− 10%) in the OB of dams. In addition, the level of C18:1n-9 was significantly decreased in the OB of the LOW group and enhanced in the HIGH group. Similar to the OM, the levels of SFAs, MUFAs, PUFAs and DMAs in the OB were not affected by the diets.

**Table 3:** Fatty acid composition of the dam olfactory mucosa and olfactory bulb (% of total fatty acids).

	Dam olfactory mucosa			Dam olfactory bulb		
Fatty acids	CON	LOW	HIGH	CON	LOW	HIGH
14:0	0.13 ± 0.02	0.14 ± 0.04	0.10 ± 0.01	0.09 ± 0.01	0.12 ± 0.02	0.12* ± 0.02
15:0	0.04 ± 0.01	0.04 ± 0.01	0.03 ± 0.01	0.03 ± 0.01	0.04* ± 0.01	0.04 ± 0.01
dma16:0	4.13 ± 0.11	3.79 ± 0.12	3.60 ± 0.22	3.24 ± 0.15	3.31 ± 0.26	3.63 ± 0.09
16:0	18.32 ± 0.73	17.30 ± 0.49	15.76 ± 0.83	22.51 ± 0.70	24.55* ± 0.69	22.04 ± 0.69
16:1n-9	0.30 ± 0.02	0.31 ± 0.03	0.26 ± 0.02	0.17 ± 0.01	0.20 ± 0.02	0.20 ± 0.01
16:1n-7	0.71 ± 0.08	1.18 ± 0.49	0.70 ± 0.05	0.52 ± 0.02	0.56 ± 0.06	0.69* ± 0.05
17:0	0.08 ± 0.01	0.08 ± 0.01	0.09 ± 0.01	0.10 ± 0.01	0.11 ± 0.01	0.10 ± 0.01
dma18:0	1.22 ± 0.04	1.23 ± 0.08	1.45 ± 0.16	2.60 ± 0.11	2.33 ± 0.03	2.44 ± 0.10
dma18:1n-9	0.61 ± 0.01	0.61 ± 0.05	0.73 ± 0.11	0.88 ± 0.07	0.74 ± 0.08	0.88 ± 0.05
dma18:1n-7	0.38 ± 0.01	0.39 ± 0.03	0.49 ± 0.11	1.09 ± 0.09	0.91 ± 0.12	1.06 ± 0.09
18:0	19.22 ± 0.24	18.68 ± 0.30	19.33 ± 0.31	20.10 ± 0.92	20.60 ± 1.37	18.33 ± 0.19
18:1t	0.05 ± 0.01	0.05 ± 0.01	0.05 ± 0.01	0.05 ± 0.01	0.14 ± 0.04	0.05 ± 0.01
18:1n-9	12.99 ± 0.39	13.53 ± 0.50	14.04 ± 0.60	14.51 ± 0.34	13.41* ± 0.53	15.25* ± 0.08
18:1n-7	2.76 ± 0.04	3.00 ± 0.17	2.71 ± 0.08	3.78 ± 0.27	3.64 ± 0.35	4.16 ± 0.05
18:2n-6	2.37 ± 0.07	2.21 ± 0.10	2.75 ± 0.20	0.41 ± 0.02	0.29 ± 0.04	0.55* ± 0.05
20:0	0.17 ± 0.01	0.17 ± 0.01	0.25 ± 0.07	0.21 ± 0.02	0.20 ± 0.01	0.21 ± 0.01
18:3n-6	0.07 ± 0.01	0.06 ± 0.01	0.06 ± 0.01	0.02 ± 0.01	0.04* ± 0.01	0.03 ± 0.01
20:1n-9	0.31 ± 0.04	0.36 ± 0.07	0.66 ± 0.38	0.92 ± 0.03	0.86 ± 0.05	0.80* ± 0.03
18:3n-3	0.11 ± 0.01	0.09 ± 0.01	0.21 ± 0.10	0.23 ± 0.01	0.23 ± 0.01	0.21 ± 0.01
20:2n-6	0.12 ± 0.01	0.12 ± 0.01	0.14 ± 0.01	0.08 ± 0.01	0.09 ± 0.01	0.15* ± 0.04
20:3n-9	0.78 ± 0.02	0.80 ± 0.04	0.71 ± 0.02	0.21 ± 0.01	0.22 ± 0.01	0.21 ± 0.01
22:0	0.13 ± 0.01	0.13 ± 0.01	0.20 ± 0.04	0.15 ± 0.03	0.17 ± 0.02	0.15 ± 0.01
20:3n-6	1.10 ± 0.02	1.12 ± 0.05	1.16 ± 0.06	0.38 ± 0.02	0.25* ± 0.02	0.50* ± 0.01
22:1n-9	0.52 ± 0.05	0.40* ± 0.02	0.56 ± 0.07	0.18 ± 0.05	0.08 ± 0.01	0.09 ± 0.01
20:4n-6	13.89 ± 0.48	16.72* ± 0.54	11.62* ± 0.34	9.56 ± 0.21	9.95 ± 0.10	8.59* ± 0.21
20:5n-3	1.16 ± 0.04	0.52* ± 0.02	2.52* ± 0.13	0.11 ± 0.01	0.07* ± 0.01	0.28* ± 0.04
24:0	0.21 ± 0.01	0.21 ± 0.02	0.37* ± 0.07	0.30 ± 0.09	0.34 ± 0.07	0.21 ± 0.01
24:1n-9	0.18 ± 0.01	0.19 ± 0.01	0.26 ± 0.05	0.49 ± 0.10	0.54 ± 0.04	0.57 ± 0.05
22:4n-6	0.91 ± 0.05	1.33* ± 0.06	0.73* ± 0.08	2.00 ± 0.05	2.18* ± 0.05	1.55* ± 0.05
22:5n-6	0.42 ± 0.02	1.50* ± 0.07	0.16* ± 0.01	0.36 ± 0.03	1.30* ± 0.12	0.15* ± 0.01
22:5n-3	0.54 ± 0.02	0.36* ± 0.01	1.01* ± 0.08	0.14 ± 0.01	0.07* ± 0.01	0.33* ± 0.01
22:6n-3	16.02 ± 0.80	13.40* ± 0.53	17.30 ± 0.86	14.57 ± 0.60	12.46* ± 0.49	16.48* ± 0.42
SFAs	38.31 ± 0.87	36.74 ± 0.36	36.14 ± 0.71	43.49 ± 1.74	46.13 ± 1.99	41.18 ± 0.59
MUFAs	17.83 ± 0.57	19.03 ± 1.15	19.25 ± 1.17	20.63 ± 0.56	19.42 ± 0.91	21.79 ± 0.08
PUFAs	37.51 ± 1.33	38.21 ± 0.97	38.35 ± 0.97	28.07 ± 0.89	27.15 ± 0.67	29.04 ± 0.59
DMAs	6.34 ± 0.09	6.02 ± 0.18	6.27 ± 0.30	7.80 ± 0.34	7.29 ± 0.44	8.00 ± 0.15
Total n-6 PUFAs	18.88 ± 0.57	23.05* ± 0.68	16.61* ± 0.49	12.80 ± 0.30	14.11* ± 0.28	11.52* ± 0.24
Total n-3 PUFAs	17.84 ± 0.84	14.36* ± 0.54	21.03* ± 0.79	15.06 ± 0.62	12.83* ± 0.50	17.31* ± 0.42
n-6:n-3 ratio	1.07 ± 0.04	1.62* ± 0.06	0.80* ± 0.04	0.85 ± 0.02	1.11* ± 0.04	0.67* ± 0.01

Data are expressed as the mean ± S.E.M. (n = 6–7 mice/group).

*SFAs* saturated fatty acids, *MUFAs* monounsaturated fatty acids, *PUFAs* polyunsaturated fatty acids, *DMAs* dimethyl acetals.

*Values are significantly different from the CON group (Mann–Whitney U test, p < 0.05).

### Offspring tissues

#### Lipid composition of olfactory mucosa

##### Fatty acid composition of total lipids

Compared to the CON group, the LOW diet significantly affected the levels of SFAs (− 22%), PUFAs (+ 33%) and DMAs (− 8%) in offspring OM (Table 4). The n-6 PUFA proportion was strongly enhanced (+ 73%), whereas the n-3 PUFA level was reduced by 40%. LOW offspring OM contained significantly higher C20:4n-6 and C22:5n-6. In contrast, lower C16:0 and C22:6n-3 levels were observed in the LOW group. The HIGH diet also modified the FA composition of offspring OM, which was found to contain higher PUFA (+ 33%) and lower SFA (− 22%) levels than the CON group. The n-3 PUFA level was strongly enhanced (+ 88%), but the n-6 PUFA level was not affected. The OM of the HIGH offspring had significantly higher C20:5n-3, C22:5n-3 and C22:6n-3 than the CON group. Conversely, the levels of C16:0 and C24:1n-9 were significantly reduced. Compared to the CON group, the n-6:n-3 ratio was significantly enhanced (3×) in the LOW group, whereas this parameter was reduced by a factor of 1.8 in the HIGH group.

**Table 4:** Fatty acid composition of the offspring olfactory mucosa and olfactory bulb (% of total fatty acids).

	Offspring olfactory mucosa			Offspring olfactory bulb		
Fatty acids	CON	LOW	HIGH	CON	LOW	HIGH
14:0	0.49 ± 0.07	0.12* ± 0.02	0.08* ± 0.02	0.12 ± 0.02	0.12 ± 0.02	0.07* ± 0.01
15:0	0.11 ± 0.01	0.06* ± 0.01	0.06* ± 0.01	0.03 ± 0.01	0.03 ± 0.01	0.04 ± 0.01
dma16:0	3.60 ± 0.14	3.02* ± 0.07	3.10* ± 0.12	3.01 ± 0.05	3.03 ± 0.08	2.79 ± 0.09
16:0	22.63 ± 0.48	14.05* ± 0.46	14.55* ± 0.51	18.94 ± 0.73	18.68 ± 0.71	17.40 ± 0.57
16:1n-9	0.42 ± 0.02	0.27* ± 0.01	0.25* ± 0.01	0.31 ± 0.01	0.34 ± 0.02	0.26* ± 0.01
16:1n-7	0.68 ± 0.07	0.37* ± 0.01	0.43* ± 0.02	0.46 ± 0.02	0.45 ± 0.02	0.42 ± 0.02
17:0	0.13 ± 0.01	0.11* ± 0.01	0.11* ± 0.01	0.10 ± 0.00	0.09 ± 0.01	0.09 ± 0.01
dma18:0	1.17 ± 0.11	1.25 ± 0.02	1.27 ± 0.03	2.51 ± 0.04	2.31* ± 0.04	2.52 ± 0.04
dma18:1n-9	0.70 ± 0.06	0.71 ± 0.01	0.73 ± 0.04	0.78 ± 0.02	0.78 ± 0.01	0.72 ± 0.03
dma18:1n-7	0.33 ± 0.01	0.36 ± 0.01	0.34 ± 0.01	0.49 ± 0.01	0.51 ± 0.01	0.45 ± 0.02
18:0	18.32 ± 0.98	18.42 ± 0.39	18.07 ± 0.61	19.23 ± 0.18	18.47* ± 0.22	19.26 ± 0.82
18:1t	0.09 ± 0.04	0.06 ± 0.00	0.04* ± 0.01	0.07 ± 0.01	0.07 ± 0.01	0.08 ± 0.02
18:1n-9	12.72 ± 0.26	12.33 ± 0.21	12.30 ± 0.34	13.72 ± 0.10	13.26* ± 0.07	13.72 ± 0.26
18:1n-7	2.76 ± 0.24	2.99 ± 0.06	2.87 ± 0.09	3.53 ± 0.05	3.70* ± 0.03	3.30 ± 0.18
18:2n-6	4.26 ± 0.40	3.61 ± 0.14	4.54 ± 0.22	0.82 ± 0.01	0.74* ± 0.02	0.79 ± 0.04
20:0	0.29 ± 0.03	0.20* ± 0.01	0.18* ± 0.01	0.21 ± 0.01	0.18* ± 0.01	0.22 ± 0.01
18:3n-6	0.12 ± 0.01	0.09* ± 0.01	0.11 ± 0.01	0.04 ± 0.01	0.05* ± 0.01	0.04 ± 0.01
20:1n-9	0.48 ± 0.06	0.68 ± 0.12	0.52 ± 0.06	0.60 ± 0.01	0.54* ± 0.01	0.65 ± 0.04
18:3n-3	0.16 ± 0.01	0.11* ± 0.01	0.14 ± 0.01	0.17 ± 0.01	0.15* ± 0.01	0.19 ± 0.02
20:2n-6	0.33 ± 0.04	0.42 ± 0.02	0.34 ± 0.01	0.22 ± 0.04	0.27 ± 0.01	0.23 ± 0.03
20:3n-9	0.37 ± 0.06	0.54* ± 0.07	0.31 ± 0.01	0.21 ± 0.01	0.23 ± 0.03	0.23* ± 0.01
22:0	0.36 ± 0.06	0.15* ± 0.01	0.16* ± 0.01	0.11 ± 0.01	0.08* ± 0.01	0.14 ± 0.02
20:3n-6	1.42 ± 0.04	1.68* ± 0.07	1.82* ± 0.05	0.56 ± 0.01	0.38* ± 0.01	0.71* ± 0.01
22:1n-9	1.47 ± 0.14	1.78 ± 0.08	1.96 ± 0.31	1.00 ± 0.16	0.96 ± 0.04	1.53 ± 0.54
20:4n-6	12.81 ± 0.37	21.17* ± 0.38	13.28 ± 0.23	12.12 ± 0.23	13.99* ± 0.22	11.22* ± 0.07
20:5n-3	0.54 ± 0.03	0.19* ± 0.01	1.89* ± 0.09	0.15 ± 0.01	0.11* ± 0.01	0.25* ± 0.01
24:0	0.56 ± 0.15	0.18* ± 0.01	0.26* ± 0.02	0.13 ± 0.03	0.08* ± 0.01	0.22 ± 0.09
24:1n-9	0.70 ± 0.08	0.29* ± 0.02	0.29* ± 0.01	0.11 ± 0.01	0.09* ± 0.01	0.18* ± 0.02
22:4n-6	1.17 ± 0.04	2.75* ± 0.05	1.02* ± 0.03	2.44 ± 0.07	3.42* ± 0.09	2.01* ± 0.04
22:5n-6	0.45 ± 0.03	5.94* ± 0.19	0.20* ± 0.01	0.79 ± 0.06	7.36* ± 0.29	0.21* ± 0.02
22:5n-3	0.55 ± 0.02	0.20* ± 0.01	1.18* ± 0.04	0.24 ± 0.01	0.09* ± 0.00	0.42* ± 0.02
22:6n-3	9.84 ± 0.67	5.93* ± 0.29	17.61* ± 1.09	16.83 ± 0.36	9.48* ± 0.17	19.70* ± 0.69
SFAs	42.88 ± 0.96	33.28* ± 0.86	33.46* ± 0.71	38.86 ± 0.83	37.73 ± 0.55	37.44 ± 1.18
MUFAs	19.32 ± 0.51	18.76 ± 0.37	18.65 ± 0.56	19.79 ± 0.17	19.39 ± 0.05	20.12 ± 0.44
PUFAs	32.01 ± 1.13	42.62* ± 0.89	42.44* ± 1.24	34.57 ± 0.70	36.25 ± 0.58	35.98 ± 0.75
DMAs	5.80 ± 0.13	5.34* ± 0.07	5.44 ± 0.17	6.79 ± 0.06	6.63 ± 0.07	6.47 ± 0.13
Total n-6 PUFAs	20.56 ± 0.60	35.66* ± 0.70	21.31 ± 0.18	16.98 ± 0.36	26.20* ± 0.57	15.21* ± 0.09
Total n-3 PUFAs	11.08 ± 0.69	6.42* ± 0.30	20.82* ± 1.16	17.38 ± 0.37	9.83* ± 0.17	20.54* ± 0.73
n-6:n-3 ratio	1.88 ± 0.08	5.60* ± 0.23	1.04* ± 0.06	0.98 ± 0.01	2.67* ± 0.08	0.74* ± 0.02

Data are the mean ± S.E.M. (n = 6 mice/group).

*SFAs* saturated fatty acids, *MUFAs* monounsaturated fatty acids, *PUFAs* polyunsaturated fatty acids, *DMAs* dimethyl acetals.

*Values are significantly different from the CON group (Mann–Whitney U test, p < 0.05).

##### Phospholipid classes and molecular species

**Figure 1:**
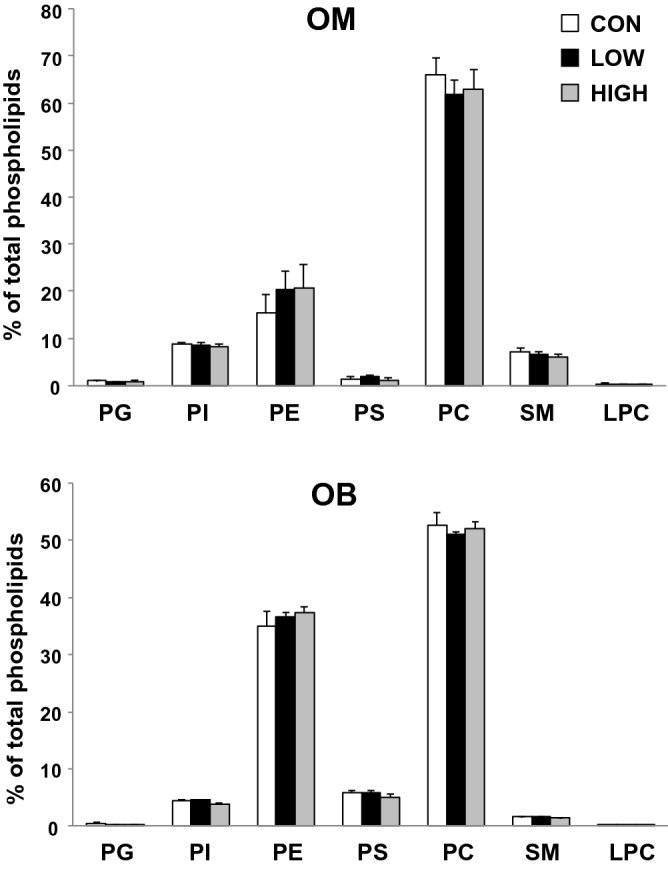
Phospholipid content of the olfactory mucosa (OM) and olfactory bulb (OB) of offspring. Data are expressed as the mean ± S.E.M. (n = 6 mice/group). The experimental groups (LOW and HIGH) were compared to the control group (CON) using the non-parametric Mann–Whitney *U* test. No significant difference was observed (p values > 0.05). *PG* Phosphatidylglycerol, *PI* phosphatidylinositol, *PE* phosphatidylethanolamine, *PS* phosphatidylserine, *PC* phosphatidylcholine, *SM* sphingomyelin, *LPC* lysophosphatidylcholine.

Analysis of PL classes by HPLC-Corona-CAD showed that PC is the most abundant PL class in offspring OM and that the PL profile is not affected by the experimental diets (Fig. 1).

**Figure 2:**
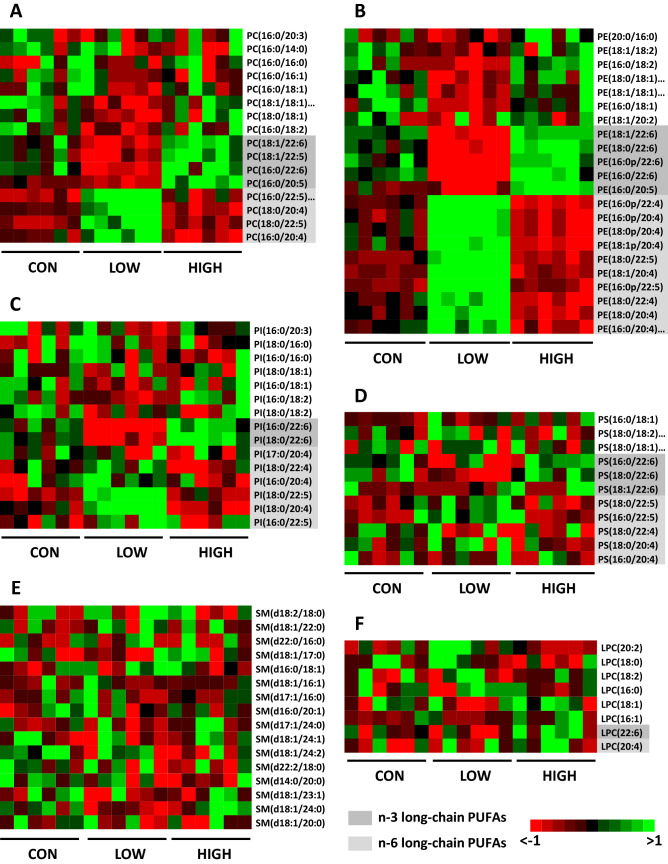
Heat maps of phospholipid molecular species in the offspring olfactory mucosa as a function of the dietary interventions (CON, LOW and HIGH diets). (**A**) Phosphatidylcholine (PC), (**B**) phosphatidylethanolamine and plasmenyl-ethanolamine (PE and PlsE), (**C**) phosphatidylserine (PS), (**D**) phosphatidylinositol (PI), (**E**) sphingomyelin (SM) and (**F**) lysophosphatidylcholine (LPC). A variability threshold (interquartile range < 0.25) was applied prior to heat map analyses. All raw data can be found in Supplementary Tables S1–S6 online.

The influence of diets on the molecular species profile of each PL class was then studied in offspring OM. For this purpose, structural analyses by mass spectrometry were performed to identify the esterified FA moieties in each PL class. Figure 2 shows heat maps based on the PL molecular species detected in offspring OM from each of the different dietary groups. The raw data are available in Supplementary Tables S1–S6. The heat map of PC species (Fig. 2A) clearly shows that the LOW and HIGH diets induced opposite effects. Compared to the CON group, an increase in n-6 PUFA-containing species and a decrease in n-3 PUFA-containing species were observed in the PC class from the LOW group, whereas greater amounts of n-3 PUFA-containing species and lower amounts of n-6 PUFA-containing species were detected in the HIGH group. The experimental diets did not modify the levels of the main molecular species found in PC (PC 16:0/18:1 and PC 16:0/16:0). The heat map of PE and plasmenyl-ethanolamine (PlsE) species detected in offspring OM (Fig. 2B) shows that the concentrations of various PE + PlsE species were strongly modified by the experimental diets. The pattern observed in this PL class is similar to that identified in the PC class. The LOW diet led to significant increases in numerous n-6 PUFA-containing species and significant decreases in n-3 PUFA-containing species in the PE/PlsE class. Conversely, high amounts of n-3 PUFA-containing species and reduced amounts of n-6 PUFA-containing species were detected in the HIGH group. Regarding the PI class, substantial modifications were induced by the LOW diet (Fig. 2C). This diet provoked a significant increase in PI 18:0/20:4 and PI 18:0/22:5 as well as a decrease in PI 16:0/22:6 and PI 18:0/22:6. The HIGH diet moderately changed the PI molecular composition: only the percentage of PI 18:0/20:4 was significantly reduced by this diet. Similarly, the LOW diet was found to affect the concentrations of a few PS species (increase in PS 16:0/22:5 and decrease in PS 18:0/22:6) (Fig. 2D), while the HIGH diet did not induce a significant change in PS molecular species. Finally, analysis of sphingomyelin (SM) species indicated that the LOW diet significantly decreased the percentage of SM d18:1/24:0 and/or SM d18:0/24:1 and/or SM d16:0/26:1 as well as SM d18:0/26:2 (Fig. 2E). Neither diet influenced the levels of lysophosphatidylcholine (LPC) molecular species (Fig. 2F).

#### Lipid composition of the olfactory bulb

##### Fatty acid composition of total lipids

When compared to the CON group, the percentage of n-6 PUFAs was greatly enhanced in the OB from offspring fed the LOW diet (+ 55%) (Table 4). A substantial increase in C22:5n-6 and C20:4n-6 occurred. In contrast, a strong decrease in n-3 PUFAs was observed (− 43%). C22:5n-3 and C22:6n-3 were markedly decreased. The HIGH diet led to a significant increase in n-3 PUFAs (+ 18%), whereas a decrease in n-6 PUFAs (− 9%) was observed. The main modifications included a decrease in C22:5n-6 and increases in C20:5n-3, C22:5n-3 and C22:6n-3.

##### Phospholipid classes and molecular species

Analysis of the OB lipid extracts by LC-Corona showed that PC and PE are the most abundant PL classes in this matrix. The experimental diets did not modify the PL profile of the offspring OB (Fig. 1).

**Figure 3:**
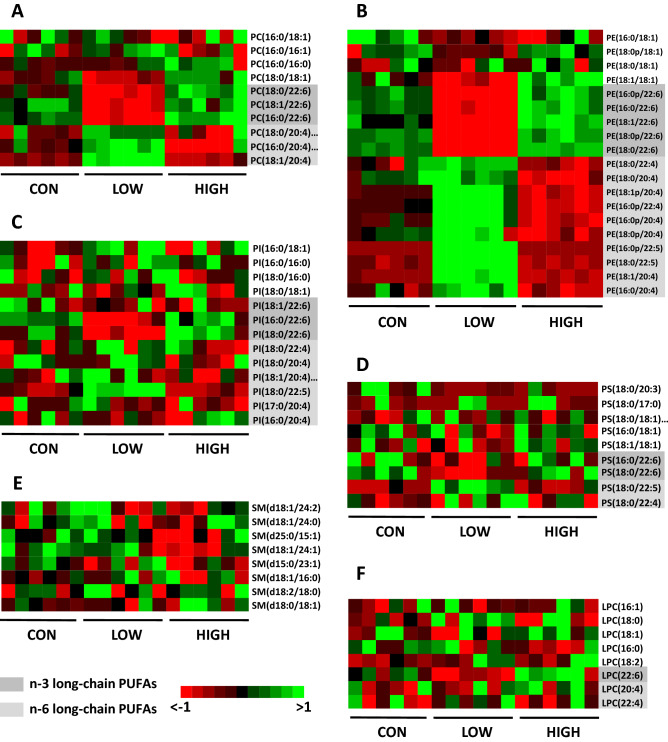
Heat maps of phospholipid molecular species in the offspring olfactory bulb as a function of the dietary interventions (CON, LOW and HIGH diets). (**A**) Phosphatidylcholine (PC), (**B**) phosphatidylethanolamine and plasmenyl-ethanolamine (PE and PlsE), (**C**) phosphatidylserine (PS), (**D**) phosphatidylinositol (PI), (**E**) sphingomyelin (SM) and (**F**) lysophosphatidylcholine (LPC). A variability threshold (interquartile range < 0.25) was applied prior to heat map analyses. All raw data can be found in Supplementary Tables S7–S12 online.

Regarding the composition of PL molecular species, the heat maps presented in Fig. 3 show striking similarities between the effects observed in the OB and those noticed in the OM (Fig. 2). In the PC and PE/PlsE classes of the offspring OB (Fig. 3A, B), the LOW diet induced an increase in various n-6 PUFA-containing species and provoked a decrease in n-3 PUFA-containing species. In contrast, the HIGH diet significantly enhanced the levels of several n-3 PUFA-containing species and reduced the levels of n-6 PUFA-containing species. It is noteworthy that, as observed in the OM, the levels of the main molecular species detected in the OB PC (PC 16:0/18:1 and PC 16:0/16:0) were not affected by the experimental diets. The LOW diet induced some significant changes in the PI and PS classes (Fig. 3C, D). A decrease in species containing C22:6n-3 as well as an increase in species containing C22:5n-6 were observed in this group. The HIGH diet did not appreciably modify the molecular profiles of PI and PS. Lastly, a small number of modifications were induced by the HIGH diet in the SM class (decrease in SM d15:0/23:1 and SM d18:1/24:1) (Fig. 3E) and by the LOW diet in the LPC class (increase in LPC 18:0 and decrease in LPC 22:6) (Fig. 3F).

## Discussion

Given the low rate of conversion of ALA into n-3 long-chain PUFAs^7^, consuming n-3 long-chain PUFAs has been widely recommended^22–24^. Moreover, the worldwide diversity of dietary intakes of n-3 FAs influences the tissue composition of n-3 long-chain PUFAs. Indeed, considerable variability in the circulating levels of n-3 PUFAs has been reported across countries^25^. The highest amounts were reported in countries and regions known to have the highest n-3 dietary consumption: Greenland, Japan, Korea and Scandinavia. In contrast, low n-3 PUFA levels were recorded in countries such as the USA and Canada with a low n-3 intake that is characteristic of so-called Western diets, suggesting n-3 deficiency in those populations. Low dietary intake of n-3 PUFAs could increase the risk of adverse health outcomes.

In the present study, our objective was to investigate the effect of feeding pregnant and lactating mice diets containing adequate LA content (1.83% of energy^26^) but either deficient in ALA or supplemented in n-3 long-chain-PUFAs on FA profiles in the dam’s milk and in olfactory tissues of dams and weaning pups. Our results revealed that perinatal consumption of these diets elicited significant changes in the total FA profiles and PL composition of the offspring OM and OB.

In adult rodent OM, PC and, to a lesser extent, PE are the main PL classes^17,27^. Plasmalogen derivatives of these two classes, PlsC and PlsE, were also detected in this tissue^17^. The analyses performed in weaning mice OM gave data that are consistent with these observations and indicated that the experimental diets did not modify the relative proportions of PL classes. However, consumption of n-3 PUFA unbalanced diets during pregnancy and lactation elicited qualitative changes in the FA profiles of the different PL classes in offspring OM, including those of plasmalogen subclasses. To summarize, feeding a low n-3 ALA diet led to a marked reduction in numerous DHA-containing species with a concomitant increase in AA-containing species, whereas consumption of a high n-3 PUFA diet reduced the prevalence of n-6 PUFA-containing species that was balanced by species containing n-3 long-chain PUFAs in PC and PE classes. These findings are in accordance with observations made in other neuronal tissues. N-3 PUFA deficiency was found to increase C22:4n-6-containing species and sometimes C22:5n-6-containing species in rodent brain PLs^28–30^. Most often, these changes were observed in animals fed n-3 PUFA-depleted diets containing high amounts of LA. In the present study, it is important to emphasize that the LOW diet contained an adequate level of LA. On the other hand, dietary supplementation with n-3 PUFAs increases the incorporation of these FAs into brain PLs at the expense of n-6 PUFA-containing species^31,32^. Similar effects were reported in the mammalian retina^33–37^. This phenomenon, which is termed “reciprocal replacement”^38^, is due to the competition between the precursors of the n-3 and n-6 PUFA families for elongation and desaturation enzymes (particularly for the first Δ6-desaturation step, which is considered the rate-limiting step)^39^. Competition might also occur during the de-esterification/re-esterification processes catalysed by enzymes such as long-chain acyl-CoA synthetases, phospholipases A2 and lysophospholipid acyltransferases. A complete replacement of DHA with C22:5n-6 has been observed in the rodent brain and retina in adulthood, but this would not be the case during postnatal development. Indeed, in addition to C22:5n-6, other n-6 PUFAs, such as C20:4n-6 and C22:4n-6, also contribute to replacing DHA in the brain and retina during the first weeks of life^40,41^. In the present study, we observed that C22:5n-6 replaced DHA in the OM from LOW weaning mice, but the n-3 PUFA-deficient diet also induced a significant increase in C20:4n-6 and C22:4n-6. These results are in line with observations made in the brain and retina during neonatal development.

In accordance with previous studies that examined the effects of n-3 PUFA modulation in various regions of the rat brain, including the OB^18–20^, we observed that the LOW and HIGH maternal diets induced significant changes in PUFA profiles in the OB from weaning mice. However, our data show that the deficiency in n-3 PUFAs elicited significant increases in C22:5n-6, C20:4n-6 and C22:4n-6, while only the C22:5n-6 level was enhanced in the rat OB. These qualitative discrepancies might result from developmental differences because in previous studies, FA analyses were performed in rats fed altered diets from conception until adulthood or during two generations. Differences in the diet formulation could also explain such divergences.

It is also worth mentioning that in the OM from weaning mice, both diets (LOW and HIGH) were found to significantly increase the level of total PUFAs and to decrease the SFA content. Such an effect was not observed in the OB of these animals. One might speculate that the OM is particularly sensitive to FA unbalanced diets and that accordingly, the in situ PUFA synthesis pathways and/or PUFA cellular input are upregulated in response to unbalanced states.

Neurodevelopmental processes take place from the embryonic period through adolescence. This period is particularly sensitive to environmental disturbances, which can lead to neurological dysfunctions^42^. The maternal diet is a factor that can have important consequences on offspring. Animal studies have shown that altered PUFA status during pregnancy and early postnatal life lead to neural, visual and behavioural deficits in offspring^9^. In humans, several epidemiological studies have suggested that maternal supplementation with n-3 PUFAs either during pregnancy or lactation can have beneficial effects on the cognitive development and visual function of infants^11^. Since both the OM and OB are neuronal tissues, it can therefore be hypothesized that consuming a maternal diet with improperly balanced PUFA composition might impact the olfactory system. Olfactory discrimination deficits have been reported in rats fed n-3 PUFA-depleted diets^18,43^. The olfactory regions affected by these diets as well as the possible mechanisms underlying these effects remain to be elucidated. Nevertheless, our study suggests that depletion of n-3 PUFAs in the OM and OB could be linked to the olfactory alterations observed in rats fed n-3 PUFA-depleted diets.

The modifications observed in the FA profiles of most PL classes in the OM and OB may have significant physiological consequences because of the importance of PL in shaping the membranes and regulating a wide range of cellular processes such as cell signalling and apoptosis. To date, there is no biochemical evidence that PLs play a functional role in olfactory signal transduction at the peripheral level. However, some observations support this idea. First, a member of the phospholipid flippase family has been shown to be essential for odourant receptor responses in *Drosophila melanogaster*^44,45^, suggesting that the PL composition of the OSN plasma membrane is an important factor for olfactory signalling. A number of studies also demonstrated that DHA-rich retinal PLs enhance the activity of rhodopsin, a receptor that belongs to the same G-protein-coupled receptor class as odourant receptors^46–53^. Specific interactions between DHA and rhodopsin improve the stability of the receptor ^54^. Finally, phosphoinositides, which are PI phosphorylated derivatives, participate in olfactory signal transduction in OM neurons^55,56^. Phosphatidylinositol(4,5)bisphosphate (PIP2) is the precursor of inositol(1,4,5)trisphosphate (IP3), a Ca^2+^ channel activator that plays a role in olfactory signal transduction in addition to the canonical cAMP signalling pathway^55,57^. In addition, phosphoinositide-3-kinase-dependent mechanisms have been implicated in the inhibition of olfactory transduction in mammalian OSNs^58,59^. In the present study, substantial modifications of the PI profile were observed in the OM from weaning mice exposed to n-3 PUFA unbalanced diets during pregnancy and lactation. The LOW diet caused a significant reduction in DHA-containing PI species and induced an increase in AA-containing species. One can envision that these changes could impair peripheral olfactory signalling catalysed by the PI pathway as well as the molecular interactions between DHA and odourant receptors.

There is compelling experimental evidence that PUFAs influence inflammatory cell function and inflammatory processes by a variety of mechanisms^2,60^. In contrast to the pro-inflammatory actions of several members of the n-6 PUFA family, the major n-3 PUFAs are capable of partly impeding a wide number of inflammation-related events. They can act either directly or after transformation into acting bioactive metabolites^61^. N-3 PUFAs exert anti-inflammatory properties in numerous tissues, especially in nervous tissues. In the brain, DHA and specific metabolites, such as neuroprotectin D1, have been shown to reduce neurodegeneration processes, suggesting that these PUFAs may prevent ageing and neurodegenerative diseases^60^. Several clinical and pre-clinical studies have also shown the neuroprotective effects of n-3 PUFAs in a number of ocular diseases, including glaucoma or optic neuropathy^62,63^. Along with these tissues, olfactory tissues can manifest symptoms related to inflammation that might contribute to olfactory dysfunction. Factors such as viral and bacterial infections or environmental toxins can cause inflammatory conditions in the OM and induce loss of OSNs^64,65^. The contaminating agents of the OM may spread thereafter to the OB, where inflammation can affect various cell types (neurons, astrocytes and microglia)^64,66^. Because olfactory tissues are enriched in DHA and its precursors, it is quite conceivable that n-3 PUFAs may be crucial for optimal functioning and for preventing neuroinflammation in these tissues. Our study showed that an n-3-deficient diet reduced the availability of DHA and its precursors in the OM and OB and resulted in a concomitant increase in n-6 PUFA-containing species. It could increase the prevalence of eicosanoids, the pro-inflammatory n-6 derivatives, accelerating the inflammation processes in olfactory cells. PUFA anti- or pro-inflammatory activities imply the release of FAs esterified into PLs by phospholipase A2 (PLA2) enzymes. The PLA2s most frequently involved in the cellular production of bioactive lipids are cytosolic calcium-dependent PLA2 (cPLA2), cytosolic calcium-independent PLA2 (iPLA2), and secretory PLA2 (sPLA2). These different PLA2s have been detected in rodent OM^67^ and OB^68,69^, indicating that both n-3 and n-6 PUFAs can be released from PLs to exert their physiological effects in these tissues^70^.

The present study showed that dietary n-3 PUFA deficiency during gestation and lactation induced a decrease in DHA levels in offspring olfactory tissues. Conversely, when dams were fed with an n-3 PUFA-enriched diet, increased DHA levels were recorded in olfactory tissues, especially in the OM. These observations clearly indicated that the maternal diet was capable of inducing significant changes in the FA composition of offspring olfactory tissues. Maternal dietary FAs are transferred to the foetus after crossing the placenta^71^ and are secreted in breast milk after birth^72^. Our results show that the milk FA profiles reflected the maternal diets. Similar to the LOW diet, the milk of the LOW dams contained a low n-3 FA level, whereas the milk of the HIGH group contained high amounts of n-3 PUFAs, as did the HIGH diet. However, in line with previous studies^73,74^, small amounts of n-3 long-chain PUFAs (20:5n-3, 22:5n-3 and 22:6n-3) were detected in the milk of the LOW dams. It is assumed that the n-3 long-chain PUFAs would be the result of FA mobilization from maternal fat stores^74^. Long-chain PUFA synthesis could also occur in the mammary gland during pregnancy and lactation in rodents^75^. On the other hand, the n-6 PUFA level of the HIGH group milk is similar to that of the CON group milk. This stable level indicates that there is no compensatory mechanism to counterbalance the n-3 PUFA increase in this body fluid, in contrast to the reciprocal replacement phenomena recorded in olfactory tissues. Our study exemplifies the use of Mead acid (C20:3n-9) as a marker for not only dietary essential FA intake (i.e., LA and ALA) but also derivatives of these essential FAs (i.e., C20:5n-3 and DHA)^76^. Indeed, C20:3n-9 was increased in the milk of dams fed a diet with low levels of ALA. Notably, low ALA was sufficient to increase Mead acid levels since LA was adequate in the LOW diet. In contrast, the supplementation of a diet containing adequate levels of LA and ALA with long-chain n-3 PUFAs (C20:5n-3, 22:5n-3 and DHA) significantly lowered this marker compared to conditions with adequate levels of dietary LA and ALA only.

In conclusion, this study demonstrated that perinatal exposure of mice to diets deficient in ALA or supplemented in n-3 long-chain PUFAs significantly affects the PL composition of offspring olfactory tissues. This finding reinforces previous observations showing that nutritional factors can modulate the physiology of the olfactory system in mammals^77–80^. This study also highlights the PUFA status of olfactory tissues as sensitive to modifications from the early stages of development. Disrupting PUFA status could have long-lasting effects on olfactory signalling.

## Methods

### Chemicals

Chloroform (CHCl_3_), methanol (CH_3_OH), ammonium acetate, acetonitrile (ACN) and H_2_O of liquid chromatography-mass spectrometry grade were purchased from Fisher Scientific (Illkirch, France). Commercially available PL standards were purchased from Avanti Polar Lipids INC-Coger (Paris, France). Other chemical reagents were obtained from Merck (St Quentin Fallavier, France).

### Animals

The experimental procedure was conducted in accordance with the guidelines of the European Community for the use and care of laboratory animals (2010/63/EU). It was approved by the local Ethics Committee (Comité d’Ethique de l’Expérimentation Animale Grand Campus Dijon; reference 01286.02) and the French Ministry for Research and Higher Education.

Twelve-week-old male and nulliparous female C57BL/6 mice were obtained from colonies established in the animal quarters of our laboratory (Centre des Sciences du Goût et de l’Alimentation, Dijon, France). They were maintained on a 12:12-h light–dark cycle and constant temperature conditions (20–22 °C). Female mice were placed for 5 days with males for mating and fed ad libitum with either one of the three experimental diets until weaning of their litters. Animals had free access to water.

The diets were prepared by the Experimental Foods Preparation Unit (INRAE, Jouy-en-Josas, France). They were formulated according to AIN-93G standards with 5% lipids^81^. The composition of the diets was as follows (g/kg): lipids, 50; casein, 200; corn starch, 418; sucrose, 100; maltodextrin, 132; cellulose, 50; mineral mix, 35; vitamin mix, 10; l-cystine, 3; choline bitartrate, 2.5. Commercial high-oleic sunflower oil, sunflower oil, palm oil, rapeseed oil and fish oil were mixed in various proportions to prepare the lipid blends that were incorporated into the different diets: the control diet (CON) had a ratio of n-6:n-3 of ~ 6, the low n-3 PUFA diet (LOW) had a ratio of n-6:n-3 of ~ 117, and the high n-3 PUFA diet (HIGH) had a ratio of n-6:n-3 of ~ 3 (Table 1). Compared to the CON diet, the LOW diet contained a lower level of ALA, whereas the HIGH diet contained n-3 long-chain PUFAs (C20:5n-3 (eicosapentaenoic acid), C22:5n-3 (docosapentaenoic acid) and DHA). The amount of LA in the diets accounted for 1.83% of total energy. This LA level is comparable to the level shown to be adequate to avoid n-6 PUFA deficiency symptoms in rats fed a diet containing 5% lipids (1–1.5% of total energy)^26^. The formulations of the mineral and vitamin mixes are detailed in Simon et al.^36^. All diets were stored at + 4 °C until given to the animals.

At postnatal day 15, all pups were separated from their dams for 4 h. In order to avoid hypothermia, pups’ cages were placed on a heating plate (28 × 20 cm; Gestigkeit, Düsseldorf, Germany) set at 37 °C. After 4 h of separation, dams were anaesthetized (intraperitoneal injection of ketamine (Imalgene 1000, Virbac, France; dose: 70 mg/kg in NaCl 0.9%) and xylazine (Rompun 2%, Bayer, Puteaux, France; dose: 14 mg/kg in NaCl 0.9%)) and then injected intraperitoneally with oxytocin (0.1 mL, Intervet, Unterschleissheim, Germany) to stimulate milk production. To further stimulate milk production and let-down, the females were gently massaged on the mammary regions. The 10 nipples of each female were aspirated using a Pasteur pipette to isolate a total amount of 0.5–1 mL of milk per female. Milk samples were snap-frozen and stored at − 80 °C until FA analyses. After the milking procedure, awake dams and their pups were returned to their home cage.

At weaning, the dams and the female pups were anaesthetized by i.p. injection of ketamine and xylazine (150 mg/kg and 10 mg/kg bodyweight, respectively) and exsanguinated by decapitation under anaesthesia. The OM and OB were immediately removed and snap-frozen in liquid nitrogen. They were stored at − 80 °C until further processing. Males were kept for further studies.

### Lipid extraction

Total lipids from olfactory tissues and maternal milk were extracted according to the Folch procedure^82^. Briefly, each sample was homogenized in 10 mL of a mixture of CHCl_3_/CH_3_OH (2:1, v/v) and mixed with 2 mL of 0.73% NaCl. The homogenized mixture was centrifuged (3,000 rpm, 3 min), and the lower organic phase was collected and evaporated to dryness under a stream of nitrogen. Total lipids were re-dissolved in 1 mL of CHCl_3_/CH_3_OH 1:1 (v/v) and stored under nitrogen at − 30 °C until further analyses.

### Fatty acid composition

FA methyl esters were analysed by gas chromatography-flame ionization detection as previously described by Le Bon et al.^17^.

### Separation and quantification of phospholipid classes by liquid chromatography coupled to charged aerosol detector (Corona-CAD)

The phosphorus content of the total lipid extracts was determined according to the method developed by Bartlett and Lewis, 1970^83^, as described previously^84^. The samples were then diluted to the appropriate concentration of 500 µg/mL PLs in CHCl_3_/CH_3_OH 1:1 (v/v).

PL classes were first separated by liquid chromatography (LC) under hydrophilic interaction liquid chromatography (HILIC) conditions and then detected using a CORONA ULTRA RS Charged Aerosol Detector (CCAD, Thermo Scientific, USA) to analyse complex lipids in a large range of polarity. Analysis methods including the detector parameters, instruments, elution gradient and other chromatographic separation conditions were recently published by Le Bon et al*.*^17^. This method allowed the separation and quantification of PI, PE, PS, PC, SM and LPC classes from lipid extracts of olfactory tissue samples.

### Structural analysis of phospholipids by liquid chromatography coupled to mass spectrometry

#### Chromatographic separation of phospholipids

PL classes were separated by LC under HILIC conditions using an ACCUCORE HILIC LC column (150 × 2.1 mm, 2.6 µm, Thermo Scientific, USA), as described previously^17^. High-performance LC (HPLC) separation was achieved using an ULTIMATE 3,000 LC pump equipped with a dual-gradient pump and an ULTIMATE 3,000 Autosampler from Thermo Scientific (USA). The mobile phase consisted of (A) ACN/H_2_O (95:5, v/v) containing 5 mM ammonium acetate and (B) ACN/H_2_O (50:50, v/v) containing 10 mM ammonium acetate. The solvent-gradient system of the analytical pump was as follows: 0 min 100% A, 1 min 95% A, 20 min 80% A, 23 min 65% A, 24 min 100% A and 24–39 min 100% A. The flow rate was 800 µL/min, the injection volume was 10 µL, and the column was maintained at 40 °C. The flow from LC was split using an analytical fixed flow splitter (split ratio = 1:1, post-column) from Analytical Scientific Instruments (El Sobrante, CA, USA). The liquid chromatography system was controlled by STANDARD INSTRUMENT INTEGRATION (SII) software based on Dionex Chromeleon TN 7.

#### Characterization of phospholipid species

The ORBITRAP FUSION TRIBRID mass spectrometer (Thermo Scientific, USA) was used for high-resolution analyses to identify phospholipid molecular species in olfactory tissues, as described previously^85^. This instrument was equipped with an EASY-MAX NG Ion Source (Heated Electrospray Ionization H-ESI) and was controlled by XCALIBUR 4.1 software. Positive and negative ions were monitored alternatively by switching the polarity approach with a spray voltage set to 3,500 V in positive and negative ion modes. The ORBITRAP mass analyser was used to obtain all mass spectra in full scan mode with the normal mass range and a target resolution of 240,000 (FWHM at *m/z* 200). A dynamic exclusion filter was applied with an exclusion duration of 15 s and a mass tolerance of 5 ppm. For tandem mass spectrometry (MS/MS) analyses, data-dependent mode was used for the characterization of PL species. Precursor isolation was performed in the quadrupole analyser with an isolation width of *m/z* 1.6. Higher-energy collisional dissociation (HCD) was employed for the fragmentation of PL species with an optimized stepped collision energy of 30% (± 5%). The linear ion trap was used to acquire spectra for fragment ions in data-dependent mode. The automatic gain control target was set to 2.10^4^ with a maximum injection time of 50 ms. The identification of all PL species was performed using the high-accuracy data and the information collected from fragmentation spectra with the help of LIPIDSEARCH software (version 4.1.16) and the LIPID MAPS database.

#### Quantification of phospholipid species

Samples were diluted to the appropriate concentration of 25 µg/mL PLs in CHCl_3_/CH_3_OH (1:1, v/v) for analysis. The LC system under HILIC conditions was coupled to a triple quadrupole mass spectrometer (Thermo Finnigan TSQ Quantum) equipped with a standard electrospray ionization source to quantify phospholipid molecular species. Specific acquisition methods in positive and negative ion modes were optimized and used according to the studied compounds. PC, PlsC and SM species were quantified in positive ion mode by precursor ion scanning of *m/z* 184 amu, which corresponds to the choline head group. PE species lose their ethanolamine phosphate head group as a neutral fragment of 141 Da. Therefore, neutral loss scanning of 141 Da in positive ion mode was used for the selective detection and quantification of PE. Similarly, PS species lose their serine-phosphate head group as a neutral fragment of 185 Da. Therefore, neutral loss scanning of 185 Da in positive ion mode was used to quantify PS compounds. PI species show in negative ion mode a fragment at *m/z* 241 amu identified as inositol phosphate minus one molecule of H_2_O. This fragment was used for precursor ion scanning to quantify these compounds. The quantification of PlsE was performed in negative ionization mode by multiple reaction monitoring (MRM) of the parent/fragment transition for each selected plasmalogen. The data were processed using XCALIBUR software. In addition, corrections were applied to the data for isotopic overlap. More details about the electrospray source parameters and mass spectrometer methods are available in Le Bon et al*.*^17^.

### Statistical analyses

Values are expressed as the mean ± standard error of the mean (S.E.M.). Statistical analyses were carried out using STATISTICA 12.0 (StatSoft, Inc.). The non-parametric Mann–Whitney *U* test was used to perform comparisons between the control group and the experimental groups. Differences were considered significant at p < 0.05. Heat maps were generated using XLSTAT (Addinsoft).

## Supplementary information

Supplementary file1 (PDF 689 kb)
