# Supplementary material for: Perinatal exposure to diets with different n-6:n-3 fatty acid ratios affects olfactory tissue fatty acid composition
Source: Sci Rep. 2020 Jul 1;10:10785. doi: 10.1038/s41598-020-67725-9 (PMC7329853; doi:10.1038/s41598-020-67725-9)
Supplement: Supplementary file 1 — Supplementary file1 (PDF 689 kb) [file 41598_2020_67725_MOESM1_ESM.pdf]

SUPPLEMENTARY INFORMATION

TITLE

Perinatal exposure to diets with different n-6:n-3 fatty acid ratios affects olfactory tissue fatty acid composition

AUTHORS

Spiro Khoury, Vanessa Soubeyre, Stéphanie Cabaret, Laetitia Merle, Stéphane Grégoire, Nicolas Deprêtre, David Jarriault, Xavier Grosmaitre, Lionel Bretillon, Olivier Berdeaux, Niyazi Acar, Anne Marie Le Bon*

Centre des Sciences du Goût et de l'Alimentation, AgroSup Dijon, CNRS, INRAE, Université Bourgogne Franche-Comté, F-21000 Dijon, France.

Table S1: Concentrations of individual molecular species of PC and PlsC in the offspring olfactory mucosa (% of total PC + PlsC). Data are expressed as the mean ± s.e.m. (n= 6 mice/group). *: Values are significantly different from the control group CON (Mann-Whitney U test; $p < 0.05$).

**Molecular species**	**PC/PlsC species (sum composition)**	**Ion precursor *m/z* 184 [M+H]$^+$**	**CON**		**LOW**		**HIGH**	
			**mean**	**sem**	**mean**	**sem**	**mean**	**sem**
**PC(15:0/14:0)**	PC 29:0	692.5	**0.07**	0.01	**0.06**	0.01	**0.05**	0.01
**PC(16:0/14:1)**	PC 30:1	704.5	**0.11**	0.01	**0.11**	0.01	**0.10**	0.01
**PC(16:0/14:0)**	PC 30:0	706.5	**1.64**	0.06	**1.59**	0.10	**1.45**	0.11
**PC(15:1/16:0)**<br>**PC(16:0p/16:0)**	PC 31:1<br>PlsC 32:0	718.5	**0.20**	0.01	**0.20**	0.02	**0.18**	0.01
**PC(15:0/16:0)**	PC 31:0	720.5	**0.31**	0.02	**0.34**	0.03	**0.31**	0.03
**PC(14:0/18:2)**	PC 32:2	730.5	**0.25**	0.02	**0.27**	0.02	**0.25**	0.03
**PC(16:0/16:1)**	PC 32:1	732.5	**2.99**	0.08	**2.98**	0.13	**2.86**	0.08
**PC(16:0/16:0)**	PC 32:0	734.6	**13.57**	0.74	**13.43**	0.53	**14.13**	0.61
**PC(16:0p/18:1)**	PlsC 34:1	744.6	**0.24**	0.01	**0.25**	0.02	**0.20**	0.02
**PC(17:1/16:0)**<br>**PC(16:0p/18:0)**	PC 33:1<br>PlsC 34:0	746.6	**0.61**	0.04	**0.56**	0.02	**0.58**	0.02
**PC(14:0/20:4)**	PC 34:4	754.5	**0.22**	0.01	**0.28**	0.02	**0.18**	0.02
**PC(16:1/18:2)**	PC 34:3	756.5	**0.46**	0.03	**0.32***	0.02	**0.39**	0.04
**PC(16:0/18:2)**	PC 34:2	758.6	**6.56**	0.50	**4.89***	0.36	**6.60**	0.66
**PC(16:0/18:1)**	PC 34:1	760.6	**23.60**	0.72	**21.91**	0.72	**23.03**	0.47
**PC(16:0p/20:4)**	PlsC 36:4	766.6	**0.54**	0.03	**0.65**	0.04	**0.59**	0.05
**PC(15:0/20:4)**	PC 35:4	768.5	**0.36**	0.03	**0.42**	0.02	**0.22***	0.03
**PC(17:0/18:1)**	PC 35:1	774.6	**0.32**	0.02	**0.24***	0.02	**0.31**	0.02
**PC(14:0/22:6)**	PC 36:6	778.5	**0.15**	0.02	**0.07***	0.01	**0.20**	0.03
**PC(16:0/20:5)**	PC 36:5	780.5	**0.77**	0.05	**0.38***	0.04	**1.88***	0.20
**PC(16:0/20:4)**	PC 36:4	782.6	**8.68**	0.34	**11.92***	0.59	**7.29***	0.28
**PC(16:0/20:3)**	PC 36:3	784.6	**3.11**	0.15	**2.92**	0.18	**3.17**	0.17
**PC(18:1/18:1)**<br>**PC(18:0/18:2)**	PC 36:2	786.6	**6.09**	0.23	**5.19***	0.12	**5.92**	0.22
**PC(18:0/18:1)**	PC 36:1	788.6	**5.88**	0.17	**5.03***	0.15	**5.99**	0.41
**PC(16:0p/22:6)**	PlsC 38:6	790.6	**0.35**	0.04	**0.21**	0.07	**0.42**	0.07
**PC(15:0/22:6)**<br>**PC(18:1p/20:4)**	PC 37:6<br>PlsC 38:5	792.5	**0.25**	0.05	**0.28**	0.01	**0.28**	0.05
**PC(18:0p/20:4)**	PlsC 38:4	794.6	**0.27**	0.04	**0.44***	0.04	**0.23**	0.03
**PC(16:1/22:6)**	PC 38:7	804.5	**0.19**	0.02	**0.10***	0.02	**0.23**	0.02
**PC(16:0/22:6)**	PC 38:6	806.6	**5.66**	0.44	**2.37***	0.27	**7.05**	0.70
**PC(16:0/22:5)**<br>**PC(18:0/20:5)**	PC 38:5	808.6	**2.40**	0.12	**4.55***	0.18	**2.72**	0.27
**PC(18:0/20:4)**	PC 38:4	810.6	**6.93**	0.31	**11.12***	0.77	**5.11***	0.41
**PC(18:0/20:3)**	PC 38:3	812.6	**1.86**	0.12	**1.86**	0.05	**1.72**	0.13
**PC(18:2/21:6)**	PC 38:1	816.5	**0.36**	0.04	**0.28**	0.05	**0.25**	0.03
**PC(17:0/22:6)**	PC 39:6	820.6	**0.16**	0.01	**0.15**	0.02	**0.17**	0.02
**PC(20:4/20:4)**	PC 40:8	830.6	**0.15**	0.02	**0.10***	0.01	**0.16**	0.02
**PC(18:1/22:6)**	PC 40:7	832.6	**1.08**	0.10	**0.54***	0.06	**1.44***	0.11
**PC(18:1/22:5)**	PC 40:6	834.6	**2.59**	0.17	**1.80***	0.17	**3.34***	0.20
**PC(18:0/22:5)**	PC 40:5	836.6	**0.49**	0.05	**1.38***	0.13	**0.47**	0.05
**PC(18:0/22:4)**	PC 40:4	838.6	**0.30**	0.03	**0.55***	0.05	**0.21***	0.02
**PC(20:4/22:6)**	PC 42:10	854.6	**0.04**	0.01	**0.02**	0.01	**0.05**	0.01
**PC(22:4/20:4)**	PC 42:8	858.6	**0.06**	0.01	**0.09**	0.02	**0.07**	0.01
**PC(20:2/22:5)**	PC 42:7	860.6	**0.06**	0.01	**0.07**	0.01	**0.06**	0.01
**PC(20:1/22:5)**	PC 42:6	862.6	**0.05**	0.01	**0.04**	0.01	**0.06**	0.01
**PC(22:6/22:6)**	PC 44:12	878.6	**0.03**	0.01	**0.01***	0.01	**0.04**	0.01
**PC(22:5/22:6)**	PC44:11	880.6	**0.00**	0.00	**0.02***	0.01	**0.02**	0.01

Table S2: Concentrations of individual molecular species of PE and PlsE in the offspring olfactory mucosa (% of total PE + PlsE). Data are expressed as the mean ± s.e.m. (n= 6 mice/group). *: Values are significantly different from the control group CON (Mann-Whitney U test; $p < 0.05$).

**Molecular species**	**PE/PlsE species (sum composition)**	**PE: neutral loss *m/z* 141<br>PlsE: MRM transition**	**CON**		**LOW**		**HIGH**	
			**mean**	**sem**	**mean**	**sem**	**mean**	**sem**
**PE(18:1/14:1)**	PE 32:2	688.5	**0.03**	0.03	**0.02**	0.01	**0.04**	0.04
**PE(16:0/16:1)**	PE 32:1	690.5	**0.23**	0.06	**0.19**	0.05	**0.25**	0.06
**PE(16:0/16:0)**	PE 32:0	692.5	**0.18**	0.07	**0.22**	0.05	**0.17**	0.04
**PE(16:1/18:2)**	PE 34:3	714.5	**0.21**	0.14	**0.09***	0.02	**0.19**	0.10
**PE(16:0/18:2)**	PE 34:2	716.5	**1.83**	0.44	**1.22***	0.19	**2.24**	0.46
**PE(16:0/18:1)**	PE 34:1	718.5	**4.26**	0.56	**3.12***	0.26	**4.17**	0.77
**PE(16:0/20:5)**	PE 36:5	738.5	**0.64**	0.13	**0.41**	0.17	**1.20***	0.13
**PE(16:0/20:4)<br>PE(18:2/18:2)**	PE 36:4	740.5	**7.31**	0.25	**8.45***	0.60	**6.26***	0.63
**PE(18:1/18:2)**	PE 36:3	742.5	**2.16**	0.42	**1.49***	0.50	**2.21**	0.86
**PE(18:1/18:1)<br>PE(16:0/20:2)**	PE 36:2	744.6	**6.46**	1.42	**4.14***	0.74	**5.97**	1.03
**PE(18:0/18:1)<br>PE(16:0/20:1)**	PE 36:1	746.6	**3.36**	0.55	**2.44***	0.39	**3.44**	0.56
**PE(20:0/16:0)**	PE 36:0	748.6	**0.45**	0.18	**0.29**	0.12	**0.69**	0.29
**PE(16:1/22:6)**	PE 38:7	762.5	**0.59**	0.11	**0.41***	0.09	**0.61**	0.20
**PE(16:0/22:6)**	PE 38:6	764.5	**14.76**	1.53	**6.46***	0.67	**17.83**	2.70
**PE(18:1/20:4)**	PE38:5	766.5	**6.35**	0.34	**12.37***	0.38	**5.73**	0.59
**PE(18:0/20:4)**	PE 38:4	768.6	**23.24**	1.01	**28.38***	1.33	**20.33***	1.30
**PE(18:1/20:2)**	PE 38:3	770.6	**1.36**	0.28	**1.29**	0.47	**1.34**	0.28
**PE(18:0/20:1)**	PE 38:1	774.6	**0.19**	0.10	**0.13**	0.06	**0.26**	0.11
**PE(18:2/22:6)**	PE 40:8	788.5	**0.46**	0.17	**0.42**	0.11	**0.60**	0.07
**PE(18:1/22:6)**	PE 40:7	790.5	**4.20**	0.31	**2.35***	0.23	**4.73***	0.19
**PE(18:0/22:6)**	PE 40:6	792.6	**8.77**	0.80	**5.57***	0.37	**11.14***	0.68
**PE(18:0/22:5)**	PE 40:5	794.6	**1.80**	0.44	**5.18***	0.40	**1.35***	0.24
**PE(18:0/22:4)**	PE 40:4	796.6	**1.75**	0.25	**2.75***	0.33	**1.06***	0.18
**PE(20:1/22:5)**	PE 42:6	820.6	**0.09**	0.07	**0.17**	0.08	**0.09**	0.03
**PE(16:0p/20:4)**	160p/20:4	722 ->303	**3.31**	0.38	**4.88***	0.18	**2.74***	0.21
**PE(16:0p/20:3)**	160p/20:3	724 ->305	**0.22**	0.02	**0.31***	0.02	**0.16***	0.01
**PE(16:0p/22:6)**	160p/22:6	746 ->327	**1.36**	0.26	**0.69***	0.05	**1.55**	0.15
**PE(18:1p/20:4)**	181p/20:4	748 -> 303	**0.98**	0.15	**1.36***	0.06	**0.72***	0.05
**PE(16:0p/22:5)**	160p/22:5	748 -> 329	**0.40**	0.05	**1.14***	0.07	**0.36**	0.16
**PE(18:0p/20:4)**	180p/20:4	750 -> 303	**1.53**	0.14	**2.04***	0.07	**1.24***	0.11
**PE(16:0p/22:4)**	160p/22:4	750 -> 331	**0.88**	0.09	**1.73***	0.10	**0.54***	0.03
**PE(18:1p/22:6)**	181p/22:6	772 -> 327	**0.31**	0.05	**0.15***	0.01	**0.34**	0.03
**PE(18:0p/22:6)**	180p/22:6	774 -> 327	**0.34**	0.08	**0.16***	0.02	**0.41**	0.05

Table S3: Concentrations of individual molecular species of PI in the offspring olfactory mucosa (% of total PI). Data are expressed as the mean ± s.e.m. (n= 6 mice/group). *: Values are significantly different from the control group CON (Mann-Whitney U test; $p < 0.05$).

Molecular species	PI species (sum composition)	Ion precursor *m/z* 271 (InsP-H2O) $[M-H]^-$	CON		LOW		HIGH	
			mean	sem	mean	sem	mean	sem
**PI(16:0/14:0)**	PI 30:0	781.5	**0.20**	0.08	**0.25**	0.05	**0.15**	0.04
**PI(16:0/16:0)**	PI 32:0	809.5	**5.65**	0.65	**5.23**	0.66	**4.56**	0.50
**PI(16:0/18:2)**	PI 34:2	833.5	**0.81**	0.16	**0.45**	0.10	**1.13**	0.22
**PI(16:0/18:1)**	PI 34:1	835.5	**4.77**	0.41	**3.40***	0.43	**4.27**	0.70
**PI(18:0/16:0)**	PI 34:0	837.5	**4.38**	0.59	**4.96**	0.50	**4.55**	0.43
**PI(16:0/20:4)**	PI 36:4	857.5	**9.47**	0.70	**10.18**	0.30	**9.11**	0.40
**PI(16:0/20:3)**	PI 36:3	859.5	**1.17**	0.35	**0.82**	0.26	**1.00**	0.12
**PI(18:0/18:2)**	PI 36:2	861.5	**1.69**	0.17	**1.10***	0.17	**1.26**	0.33
**PI(18:0/18:1)**	PI 36:1	863.6	**1.49**	0.23	**1.11**	0.30	**1.39**	0.26
**PI(18:0p/20:4)**	PI 38:4	869.6	**0.25**	0.05	**0.41**	0.10	**0.12**	0.04
**PI(17:0/20:4)**	PI 37:4	871.5	**0.37**	0.07	**0.30**	0.11	**0.28**	0.13
**PI(16:0/22:6)**	PI 38:6	881.5	**6.91**	0.55	**3.15***	0.18	**8.80**	0.77
**PI(16:0/22:5)**	PI 38:5	883.5	**3.41**	0.43	**4.92**	0.56	**3.93**	0.49
**PI(18:0/20:4)**	PI 38:4	885.5	**44.06**	0.82	**51.72***	0.91	**39.92***	0.93
**PI(18:0/22:6)**	PI 40:6	909.5	**14.01**	0.81	**6.25***	0.86	**18.15***	0.92
**PI(18:0/22:5)**	PI 40:5	911.6	**0.51**	0.17	**4.39***	0.28	**0.94**	0.34
**PI(18:0/22:4)**	PI 40:4	913.6	**0.86**	0.19	**1.35**	0.23	**0.44**	0.17

Table S4: Concentrations of individual molecular species of PS in the offspring olfactory mucosa (% of total PS). Data are expressed as the mean ± s.e.m. (n= 6 mice/group). *: Values are significantly different from the control group CON (Mann-Whitney U test; $p < 0.05$).

**Molecular species**	**PS species (sum composition)**	**Neutral loss *m/z* 185 [M+H]$^+$**	**CON**		**LOW**		**HIGH**	
			**mean**	**sem**	**mean**	**sem**	**mean**	**sem**
**PS(16:0/18:1)**	PS 34:1	762.5	**2.60**	0.62	**5.17**	2.59	**3.36**	1.10
**PS(16:0/20:4)**	PS 36:4	784.5	**1.20**	0.47	**1.22**	0.48	**1.00**	0.53
**PS(18:0/18:2)**	PS 36:2	788.5	**6.61**	1.15	**7.02**	1.98	**6.59**	2.36
**PS(18:1/18:1)**								
**PS(18:0/18:1)**	PS 36:1	790.6	**29.28**	4.56	**28.72**	6.75	**28.00**	4.17
**PS(16:0/20:1)**								
**PS(16:0/22:6)**	PS 38:6	808.5	**16.54**	1.88	**8.29**	3.12	**17.32**	2.10
**PS(16:0/22:5)**	PS 38:5	810.5	**2.89**	1.96	**11.47***	2.12	**3.68**	2.27
**PS(18:0/20:4)**	PS 38:4	812.5	**15.78**	1.43	**20.43**	3.70	**12.99**	1.99
**PS(18:1/22:6)**	PS 40:7	834.5	**0.57**	0.35	**0.50**	0.27	**1.56**	0.69
**PS(18:0/22:6)**	PS 40:6	836.5	**17.46**	2.82	**6.59***	1.78	**20.87**	3.28
**PS(18:0/22:5)**	PS 40:5	838.6	**2.91**	1.07	**7.61**	2.04	**2.38**	1.18
**PS(18:0/22:4)**	PS 40:4	840.6	**4.16**	0.54	**2.97**	1.53	**2.24**	1.15

Table S5: Concentrations of individual molecular species of SM in the offspring olfactory mucosa (% of total SM). Data are expressed as the mean ± s.e.m. (n= 6 mice/group). *: Values are significantly different from the control group CON (Mann-Whitney U test; $p < 0.05$).

**Molecular species**	**SM species (sum composition)**	**Ion precursor *m/z* 184 [M+H]+**	**CON**		**LOW**		**HIGH**	
			**mean**	**sem**	**mean**	**sem**	**mean**	**sem**
**SM(d17:1/16:0)**	SM 33:1	689.6	**0.35**	0.11	**0.45**	0.30	**0.27**	0.08
**SM(d18:1/16:1)**	SM 34:2	701.6	**0.41**	0.15	**0.72**	0.37	**0.46**	0.06
**SM(d16:0/18:1)**	SM 34:1	703.6	**30.41**	2.80	**36.79**	4.38	**31.49**	3.07
**SM(d14:0/20:0)**	SM 34:0	705.6	**2.51**	0.37	**1.48**	0.53	**1.22**	0.56
**SM(d18:1/17:0)**	SM 35:1	717.6	**0.36**	0.11	**0.34**	0.12	**0.46**	0.08
**SM(d18:2/18:0)**	SM 36:2	729.6	**0.26**	0.06	**0.36**	0.08	**0.26**	0.07
**SM(d16:0/20:1)**	SM 36:1	731.6	**13.29**	0.68	**13.56**	0.94	**13.41**	0.33
**SM(d14:1/23:0)**	SM 37:1	745.6	**0.21**	0.04	**0.16**	0.07	**0.24**	0.07
**SM(d18:2/20:2)**	SM 38:4	753.6	**0.08**	0.03	**0.09**	0.04	**0.06**	0.02
**SM(d14:1/24:2)**	SM 38:3	755.6	**0.03**	0.01	**0.06**	0.06	**0.02**	0.02
**SM(d18:1/20:0)**	SM 38:1	759.6	**6.01**	0.43	**5.58**	0.55	**5.86**	0.67
**SM(d22:0/16:0)**	SM 38:0	761.7	**0.29**	0.13	**0.57**	0.26	**0.30**	0.16
**SM(d15:0/24:1)**	SM 39:1	773.7	**0.12**	0.02	**0.22**	0.10	**0.20**	0.08
**SM(d18:2/22:2)**	SM 40:4	781.6	**0.05**	0.01	**0.08**	0.03	**0.06**	0.03
**SM(d22:2/18:0)**	SM 40:2	785.7	**0.71**	0.10	**0.71**	0.18	**0.52**	0.15
**SM(d18:1/22:0)**	SM 40:1	787.7	**8.78**	0.62	**10.01**	1.16	**9.53**	0.73
**SM(d18:1/23:1)**	SM 41:2	799.7	**0.30**	0.09	**0.13**	0.07	**0.24**	0.08
**SM(d17:1/24:0)**	SM 41:1	801.7	**0.49**	0.06	**0.45**	0.12	**0.70**	0.14
**SM(d18:1/24:4)**	SM 42:5	807.6	**0.13**	0.05	**0.08**	0.04	**0.11**	0.06
**SM(d20:0/22:4)**	SM 42:4	809.7	**0.14**	0.06	**0.04**	0.02	**0.02***	0.02
**SM(d18:1/24:2)**	SM 42:3	811.7	**2.63**	0.24	**2.07**	0.48	**1.82**	0.35
**SM(d18:1/24:1)**	SM 42:2	813.7	**20.06**	2.49	**17.75**	2.43	**19.61**	1.48
**SM(d18:1/24:0)**<br>**SM(d18:0/24:1)**<br>**SM(d16:0/26:1)**	SM 42:1	815.7	**12.04**	0.69	**8.27***	1.05	**12.97**	1.31
**SM(d18:0/26:2)**	SM 44:2	841.7	**0.32**	0.09	**0.04***	0.02	**0.17**	0.07

Table S6: Concentrations of individual molecular species of LPC in the offspring olfactory mucosa (% of total LPC). Data are expressed as the mean ± s.e.m. (n= 6 mice/group). *: Values are significantly different from the control group CON (Mann-Whitney U test; $p < 0.05$).

**Molecular species**	**Ion precursor m/z 184 $[M+H]^+$**	**CON**		**LOW**		**HIGH**	
		**mean**	**sem**	**mean**	**sem**	**mean**	**sem**
**LPC(16:1)**	494.3	**0.37**	0.22	**0.48**	0.19	**1.53**	0.64
**LPC(16:0)**	496.3	**59.44**	2.44	**60.22**	2.27	**59.21**	1.11
**LPC(18:2)**	520.3	**2.51**	0.68	**2.46**	0.70	**2.76**	0.28
**LPC(18:1)**	522.4	**10.49**	1.10	**9.88**	1.56	**11.96**	1.10
**LPC(18:0)**	524.4	**23.63**	1.70	**22.79**	1.36	**19.18**	1.69
**LPC(20:4)**	544.3	**2.21**	0.63	**2.71**	0.68	**3.39**	0.78
**LPC(20:2)**	548.4	**0.14**	0.06	**0.44**	0.12	**0.06**	0.03
**LPC(20:1)**	550.4	**0.26**	0.17	**0.35**	0.31	**0.30**	0.14
**LPC(22:6)**	568.3	**0.95**	0.19	**0.66**	0.20	**1.60**	0.33

Table S7: Concentrations of individual molecular species of PC in the offspring olfactory bulb (% of total PC + PlsC). Data are expressed as the mean ± s.e.m. (n= 6 mice/group). *: Values are significantly different from the control group CON (Mann-Whitney U test; $p < 0.05$).

Molecular species	PC/PlsC species (sum composition)	Ion precursor m/z 184 [M+H]+	CON		LOW		HIGH	
			mean	sem	mean	sem	mean	sem
PC(16:0/14:0)	PC 30:0	**706.5**	**1.82**	0.17	**1.82**	0.04	**1.59**	0.22
PC(15:0/16:1) PC(16:0p/16:0)	PC 31:1 PlsC 32:0	**718.5**	**0.15**	0.02	**0.18**	0.01	**0.16**	0.04
PC(15:0/16:0)	PC 31:0	**720.5**	**0.35**	0.05	**0.38**	0.01	**0.31**	0.06
PC(16:1/16:1)	PC 32:2	**730.5**	**0.10**	0.02	**0.12**	0.01	**0.08***	0.01
PC(16:0/16:1)	PC 32:1	**732.5**	**3.50**	0.32	**3.72**	0.07	**3.43**	0.27
PC(16:0/16:0)	PC 32:0	**734.6**	**25.28**	0.43	**25.49**	0.25	**25.36**	3.43
PC(17:1/16:0)	PC 33:1 PlsC 34:0	**746.6**	**0.71**	0.08	**0.71**	0.01	**0.72**	0.04
PC(16:1/18:1)	PC 34:2	**758.6**	**1.62**	0.09	**1.51**	0.06	**1.45***	0.09
PC(16:0/18:1)	PC 34:1	**760.6**	**33.03**	0.67	**32.71**	0.22	**32.56**	1.18
PC(16:0p/20:4)	PlsC 36:4	**766.6**	**0.41**	0.05	**0.42**	0.07	**0.46**	0.12
PC(19:1/16:0)	PC 35:1	**774.6**	**0.29**	0.04	**0.25**	0.03	**0.29**	0.08
PC(16:0/20:4) PC(18:2/18:2)	PC 36:4	**782.6**	**5.33**	0.27	**6.08***	0.10	**4.72**	0.66
PC(18:1/18:2) PC(16:0/20:3)	PC 36:3	**784.6**	**0.65**	0.07	**0.59**	0.03	**0.78**	0.11
PC(18:1/18:1)	PC 36:2	**786.6**	**2.77**	0.15	**2.67**	0.04	**2.74**	0.20
PC(18:0/18:1)	PC 36:1	**788.6**	**8.11**	0.29	**7.70***	0.04	**8.70***	0.63
PC(16:0p/22:6)	PlsC 38:6	**790.6**	**0.20**	0.10	**0.19**	0.04	**0.23**	0.07
PC(16:1/22:6)	PC 38:7	**804.5**	**0.11**	0.01	**0.09**	0.01	**0.13**	0.03
PC(16:0/22:6)	PC 38:6	**806.6**	**3.82**	0.24	**2.01***	0.10	**4.51***	0.36
PC(18:1/20:4)	PC 38:5	**808.6**	**1.87**	0.14	**3.69***	0.09	**1.68**	0.29
PC(18:0/20:4) PC(16:0/22:4)	PC 38:4	**810.6**	**5.58**	0.33	**6.06***	0.05	**5.50**	0.79
PC(20:1/18:1)	PC 38:2	**814.6**	**0.49**	0.04	**0.48**	0.03	**0.45***	0.01
PC(18:0/20:1)	PC 38:1	**816.5**	**0.29**	0.04	**0.25**	0.01	**0.26**	0.09
PC(20:4/20:4)	PC 40:8	**830.6**	**0.08**	0.01	**0.08**	0.01	**0.07**	0.03
PC(18:1/22:6)	PC 40:7	**832.6**	**0.83**	0.09	**0.49***	0.02	**0.87**	0.12
PC(18:0/22:6)	PC 40:6	**834.6**	**1.87**	0.12	**1.45***	0.03	**2.31***	0.33
PC(18:0/22:4)	PC 40:4	**838.6**	**0.49**	0.06	**0.65***	0.03	**0.42***	0.03
PC(20:4/22:6)	PC 42:10	**854.6**	**0.07**	0.02	**0.04**	0.01	**0.09**	0.03
PC(22:4/20:4)	PC 42:8	**858.6**	**0.07**	0.02	**0.09***	0.01	**0.02***	0.00
PC(18:1/24:0)	PC 42:1	**872.7**	**0.05**	0.01	**0.03**	0.01	**0.05**	0.02
PC(22:6/22:6)	PC 44:12	**878.6**	**0.04**	0.02	**0.02***	0.01	**0.06**	0.02
PC(22:5/22:6)	PC44:11	**880.6**	**0.01**	0.01	**0.03***	0.01	**0.01**	0.01

Table S8: Concentrations of individual molecular species of PE in the offspring olfactory bulb (% of total PE + PlsE). Data are expressed as the mean ± s.e.m. (n= 6 mice/group). *: Values are significantly different from the control group CON (Mann-Whitney U test; $p < 0.05$).

Molecular species	PE/PlsE species (sum composition)	PE: neutral loss *m/z* 141 PlsE: MRM transition	CON		LOW		HIGH	
			mean	sem	mean	sem	mean	sem
**PE(15:0/15:0)**	PE 30:0	664.5	**0.01**	0.01	**0.01**	0.01	**0.01**	0.01
**PE(16:0/16:1)**	PE 32:1	690.5	**0.12**	0.03	**0.10**	0.02	**0.13**	0.05
**PE(16:0/16:0)**	PE 32:0	692.5	**0.16**	0.07	**0.23**	0.04	**0.17**	0.04
**PE(16:1/18:1)**	PE 34:2	716.5	**0.45**	0.05	**0.36***	0.08	**0.51**	0.10
**PE(16:0/18:1)**	PE 34:1	718.5	**5.16**	0.27	**4.70***	0.12	**4.78**	0.46
**PE(18:0/16:0)**	PE 34:0	720.6	**0.83**	0.09	**0.76**	0.10	**0.78**	0.09
**PE(16:1/20:4)**	PE 36:5	738.5	**0.13**	0.04	**0.16**	0.05	**0.20**	0.07
**PE(16:0/20:4)**	PE 36:4	740.5	**3.26**	0.27	**3.85***	0.26	**2.89***	0.17
**PE(18:1/18:2)**	PE 36:3	742.5	**0.40**	0.12	**0.36**	0.11	**0.60***	0.12
**PE(18:1/18:1)**	PE 36:2	744.6	**4.67**	0.40	**3.79***	0.21	**5.31***	0.30
**PE(18:0/18:1)**	PE 36:1	746.6	**3.33**	0.24	**3.35**	0.39	**3.38**	0.32
**PE(16:1/22:6)**	PE 38:7	762.5	**0.41**	0.08	**0.28***	0.05	**0.38**	0.10
**PE(16:0/22:6)**	PE 38:6	764.5	**13.43**	0.77	**7.54***	0.95	**15.01***	1.10
**PE(18:1/20:4)**	PE38:5	766.5	**3.35**	0.19	**10.08***	0.96	**2.80**	0.52
**PE(18:0/20:4)**	PE 38:4	768.6	**20.54**	0.92	**21.51**	0.90	**17.98***	0.67
**PE(20:1/18:1)**	PE 38:2	772.6	**0.14**	0.09	**0.05**	0.05	**0.17**	0.16
**PE(18:0/20:1) PE(20:0/18:1)**	PE 38:1	774.6	**0.45**	0.08	**0.33**	0.14	**0.46**	0.16
**PE(18:2/22:6)**	PE 40:8	788.5	**0.34**	0.10	**0.41**	0.10	**0.32**	0.08
**PE(18:1/22:6)**	PE 40:7	790.5	**3.98**	0.28	**2.66***	0.13	**4.74***	0.19
**PE(18:0/22:6)**	PE 40:6	792.6	**24.09**	0.48	**13.98***	0.95	**26.24***	0.57
**PE(18:0/22:5)**	PE 40:5	794.6	**1.29**	0.46	**10.17***	0.81	**0.77**	0.36
**PE(18:0/22:4)**	PE 40:4	796.6	**3.00**	0.44	**3.62***	0.23	**2.53***	0.35
**PE(20:4/22:6)**	PE 42:10	812.5	**0.25**	0.07	**0.13***	0.05	**0.21**	0.06
**PE(22:4/20:4)**	PE 42:8	816.6	**0.30**	0.06	**0.48***	0.13	**0.20***	0.03
**PE(24:5/18:0)**	PE 42:5	822.6	**0.12**	0.06	**0.29***	0.11	**0.09**	0.04
**PE(24:4/18:0)**	PE 42:4	824.6	**0.14**	0.06	**0.21**	0.09	**0.17**	0.05
**PE(22:6/22:6)**	PE 44:12	836.5	**0.17**	0.04	**0.05***	0.03	**0.25**	0.07
**PE(22:5/22:6)**	PE 44:11	838.5	**0.05**	0.03	**0.12***	0.02	**0.12***	0.06
**PE(22:4/22:6)**	PE 44:10	840.6	**0.80**	0.15	**0.70**	0.16	**0.72**	0.10
**PE(16:0p/20:4)**	160p/20:4	722 ->303	**1.43**	0.07	**1.89***	0.15	**1.17***	0.09
**PE(16:0p/20:3)**	160p/20:3	724 ->305	**0.11**	0.01	**0.14***	0.02	**0.09***	0.01
**PE(18:0p/18:1)**	180p/18:1	728 ->281	**0.89**	0.44	**0.80**	0.07	**1.08**	0.09
**PE(16:0p/22:6)**	160p/22:6	746 ->327	**1.25**	0.07	**0.74***	0.06	**1.39**	0.13
**PE(18:1p/20:4)**	181p/20:4	748 -> 303	**0.61**	0.006	**0.86***	0.037	**0.48***	0.013
**PE(16:0p/22:5)**	160p/22:5	748 -> 329	**0.23**	0.005	**1.05***	0.043	**0.21**	0.009
**PE(18:0p/20:4)**	180p/20:4	750 -> 303	**1.55**	0.017	**1.69**	0.072	**1.31***	0.035
**PE(16:0p/22:4)**	160p/22:4	750 -> 331	**1.30**	0.018	**1.82***	0.083	**0.96***	0.030
**PE(18:1p/22:6)**	181p/22:6	772 -> 327	**0.36**	0.010	**0.20***	0.008	**0.39**	0.014
**PE(18:0p/22:6)**	180p/22:6	774 -> 327	**0.92**	0.008	**0.50***	0.021	**0.99***	0.022

Table S9: Concentrations of individual molecular species of PI in the offspring olfactory bulb (% of total PI). Data are expressed as the mean ± s.e.m. (n= 6 mice/group). *: Values are significantly different from the control group CON (Mann-Whitney U test; $p < 0.05$).

Molecular species	PI species (sum composition)	Ion precursor *m/z* 271 (InsP-H2O) [M-H]⁻	CON		LOW		HIGH	
			mean	sem	mean	sem	mean	sem
**PI(16:0/16:0)**	PI 32:0	809.5	**3.44**	0.56	**4.02**	0.59	**4.29**	0.22
**PI(16:1/18:1)**	PI 34:2	833.5	**0.09**	0.04	**0.14**	0.04	**0.08**	0.04
**PI(16:0/18:1)**	PI 34:1	835.5	**1.47**	0.15	**2.24***	0.20	**1.71**	0.33
**PI(18:0/16:0)**	PI 34:0	837.5	**4.48**	0.45	**5.30**	0.41	**4.76**	0.65
**PI(16:2/20:4)**	PI 36:6	853.5	**0.01**	0.01	**0.00**	0.00	**0.01**	0.01
**PI(16:0/20:5)**	PI 36:5	855.5	**0.13**	0.04	**0.11**	0.04	**0.38***	0.06
**PI(16:0/20:4)**	PI 36:4	857.5	**11.25**	0.67	**10.91**	0.28	**11.69**	0.81
**PI(18:0/18:1)**	PI 36:1	863.6	**0.46**	0.14	**0.33**	0.08	**0.63**	0.19
**PI(17:0/20:4)**	PI 37:4	871.5	**0.51**	0.13	**0.51**	0.16	**0.32**	0.14
**PI(16:1/22:6)**	PI 38:7	879.5	**0.02**	0.01	**0.02**	0.02	**0.10**	0.06
**PI(16:0/22:6)**	PI 38:6	881.5	**3.65**	0.13	**1.53***	0.35	**3.89**	0.36
**PI(18:1/20:4)** **PI(18:0/20:5)**	PI 38:5	883.5	**8.50**	0.68	**9.66**	0.73	**7.68**	0.43
**PI(18:0/20:4)**	PI 38:4	885.5	**56.52**	0.94	**56.79**	0.71	**54.13**	1.37
**PI(20:4/20:4)**	PI 40:8	905.5	**0.10**	0.03	**0.12**	0.04	**0.14**	0.05
**PI(18:1/22:6)**	PI 40:7	907.5	**0.45**	0.14	**0.27**	0.09	**0.23**	0.10
**PI(18:0/22:6)**	PI 40:6	909.5	**7.95**	0.47	**4.18***	0.28	**9.09**	0.69
**PI(18:0/22:5)**	PI 40:5	911.6	**0.59**	0.21	**3.08***	0.23	**0.26**	0.17
**PI(18:0/22:4)**	PI 40:4	913.6	**0.38**	0.14	**0.80**	0.23	**0.61**	0.31

Table S10: Concentrations of individual molecular species of PS in the offspring olfactory bulb (% of total PS). Data are expressed as the mean ± s.e.m. (n= 6 mice/group). *: Values are significantly different from the control group CON (Mann-Whitney U test; $p < 0.05$).

Molecular species	PS species (sum composition)	Neutral loss *m/z* 185 $[M+H]^+$	CON		LOW		HIGH	
			mean	sem	mean	sem	mean	sem
**PS(16:0/18:1)**	PS 34:1	762.5	**9.53**	0.78	**7.10**	1.47	**9.54**	1.07
**PS(18:0/17:0)**	PS 35:0	778.6	**0.13**	0.09	**0.23**	0.14	**0.20**	0.10
**PS(18:1/18:1)**	PS 36:2	788.5	**10.05**	1.85	**8.97**	2.44	**11.41**	1.28
**PS(18:0/18:1)** **PS(16:0/20:1)**	PS 36:1	790.6	**17.31**	3.41	**19.94**	3.45	**18.51**	1.75
**PS(15:1/22:4)**	PS 37:5	796.5	**0.04**	0.01	**0.02***	0.00	**0.04**	0.02
**PS(16:0/22:6)**	PS 38:6	808.5	**10.35**	2.34	**4.93**	1.41	**8.77**	1.97
**PS(18:0/20:3)**	PS 38:3	814.6	**2.16**	0.88	**0.10***	0.05	**0.36***	0.30
**PS(18:0/22:6)**	PS 40:6	836.5	**44.10**	3.32	**28.57***	1.92	**42.24**	3.24
**PS(18:0/22:5)**	PS 40:5	838.6	**2.05**	1.38	**22.77***	4.01	**3.40**	1.92
**PS(18:0/22:4)**	PS 40:4	840.6	**4.26**	0.92	**7.37**	2.54	**5.53**	1.89

Table S11: Concentrations of individual molecular species of SM in the offspring olfactory bulb (% of total SM). Data are expressed as the mean ± s.e.m. (n= 6 mice/group). *: Values are significantly different from the control group CON (Mann-Whitney U test; $p < 0.05$).

**Molecular species**	**SM species (sum composition)**	**Ion precursor *m/z* 184 [M+H]$^+$**	**CON**		**LOW**		**HIGH**	
			**mean**	**sem**	**mean**	**sem**	**mean**	**sem**
**SM(d18:1/16:0)**	SM 34:1	706.5	**15.57**	0.55	**18.23**	2.20	**15.46**	2.81
**SM(d18:2/18:0)**	SM 36:2	730.5	**1.13**	0.15	**1.17**	0.36	**1.39**	0.22
**SM(d18:0/18:1)**	SM 36:1	732.5	**63.07**	0.77	**61.72**	2.55	**67.34**	3.07
**SM(d15:0/23:1)**	SM 38:1	754.5	**3.31**	0.16	**3.35**	0.52	**2.63***	0.22
**SM(d25:0/15:1)**	SM 40:1	768.5	**3.65**	0.31	**3.38**	0.46	**2.74**	0.44
**SM(d18:1/24:2)**	SM 42:3	784.6	**0.78**	0.10	**0.72**	0.16	**0.53**	0.11
**SM(d18:1/24:1)**	SM 42:2	786.6	**10.10**	0.47	**9.26**	0.62	**7.32***	0.73
**SM(d18:1/24:0)**	SM 42:1	788.6	**2.39**	0.41	**2.17**	0.52	**2.59**	0.55

Table S12: Concentrations of individual molecular species of LPC in the offspring olfactory bulb (% of total LPC). Data are expressed as the mean ± s.e.m. (n= 6 mice/group). *: Values are significantly different from the control group CON (Mann-Whitney U test; $p < 0.05$).

Molecular species	Ion precursor m/z 184 $[M+H]^+$	CON		LOW		HIGH	
		mean	sem	mean	sem	mean	sem
**LPC(16:1)**	494.3	**0.56**	0.19	**0.69**	0.23	**0.74**	0.28
**LPC(16:0)**	496.3	**58.57**	1.06	**57.02**	2.77	**52.27**	2.57
**LPC(18:2)**	520.3	**0.30**	0.14	**1.06**	0.35	**1.15**	0.55
**LPC(18:1)**	522.4	**16.11**	0.96	**14.16**	1.26	**16.86**	0.53
**LPC(18:0)**	524.4	**14.38**	0.78	**19.24***	1.61	**14.60**	2.15
**LPC(20:4)**	544.3	**5.89**	1.03	**6.10**	0.85	**7.89**	1.24
**LPC(22:6)**	568.3	**3.85**	0.48	**1.25***	0.29	**6.13**	0.99
**LPC(22:4)**	572.4	**0.34**	0.15	**0.49**	0.12	**0.36**	0.15
